# Investigating population‐scale allelic differential expression in wild populations of *Oithona similis* (Cyclopoida, Claus, 1866)

**DOI:** 10.1002/ece3.6588

**Published:** 2020-08-04

**Authors:** Romuald Laso‐Jadart, Kevin Sugier, Emmanuelle Petit, Karine Labadie, Pierre Peterlongo, Christophe Ambroise, Patrick Wincker, Jean‐Louis Jamet, Mohammed‐Amin Madoui

**Affiliations:** ^1^ Génomique Métabolique, Genoscope Institut François Jacob, CEA, CNRS, Univ Evry Université Paris‐Saclay Evry France; ^2^ Research Federation for the study of Global Ocean Systems Ecology and Evolution FR2022/Tara Oceans GO‐SEE Paris France; ^3^ CEA, Genoscope Institut de Biologie François Jacob Université Paris‐Saclay Evry France; ^4^ CNRS, Inria, IRISA – UMR 6074 Univ Rennes Rennes France; ^5^ LaMME, CNRS Université Paris‐Saclay Univ Evry Evry France; ^6^ Mediterranean Institute of Oceanology (MIO) AMU‐UTLN UM110 CNRS UMR7294, IRD UMR235 Equipe Ecologie Marine et Biodiversité (EMBIO) Université de Toulon Toulon Cedex 9 France

**Keywords:** allelic expression, Arctic seas, copepod, metagenomics, metatranscriptomics, selection, structure, *Tara* Oceans, Zooplankton

## Abstract

Acclimation allowed by variation in gene or allele expression in natural populations is increasingly understood as a decisive mechanism, as much as adaptation, for species evolution. However, for small eukaryotic organisms, as species from zooplankton, classical methods face numerous challenges. Here, we propose the concept of allelic differential expression at the population‐scale (psADE) to investigate the variation in allele expression in natural populations. We developed a novel approach to detect psADE based on metagenomic and metatranscriptomic data from environmental samples. This approach was applied on the widespread marine copepod, *Oithona similis,* by combining samples collected during the *Tara* Oceans expedition (2009–2013) and de novo transcriptome assemblies. Among a total of 25,768 single nucleotide variants (SNVs) of *O*.* similis*, 572 (2.2%) were affected by psADE in at least one population (FDR < 0.05). The distribution of SNVs under psADE in different populations is significantly shaped by population genomic differentiation (Pearson *r* = 0.87, *p* = 5.6 × 10^−30^), supporting a partial genetic control of psADE. Moreover, a significant amount of SNVs (0.6%) were under both selection and psADE (*p* < .05), supporting the hypothesis that natural selection and psADE tends to impact common loci. Population‐scale allelic differential expression offers new insights into the gene regulation control in populations and its link with natural selection.

## Graphical abstract

This research focused on analysing variation in allelic expression at population level, called here psADE. We extracted polymorphic data belonging to *Oithona similis*, a copepod, using metagenomic and metatranscriptomic data collected during the Tara Oceans expeditions. We found a link between loci targeted by psADE, population differentiation and selection and provided a functional analysis of these loci.

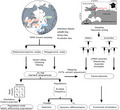

## 1 INTRODUCTION

Variation in gene expression within and between individuals or natural populations is an important mechanism for the acclimation of species (Fay & Wittkopp, 2008; Hutter, Saminadin‐Peter, Stephan, & Parsch, 2008; Li, Liu, Kim, Min, & Zhang, 2010; Whitehead, 2012). This variation can nonexclusively be driven by selective genetic factors (Zhang et al. 2008; Fraser, 2013; Sato, Makino, & Kawata, 2016) or be induced by environmental cues and gradients, for varying periods of time (Passow et al. 2017; Brown, Arias‐Rodriguez, Yee, Tobler, & Kelley, 2018). Particularly, marine species populations belonging to plankton face a large panel of physico‐chemical changes in open ocean, (Guinder & Molinero, 2013; Pelejero, Calvo, & Hoegh‐Guldberg, 2010) and gene expression variations have been observed in several studies (Lauritano, Procaccini, & Ianora, 2012; Salazar et al. 2019). However, taxonomic identification (Cepeda, Sabatini, Scioscia, Ramírez, & Viñas, 2016), DNA and mRNA extraction of small marine eukaryote species due to their complex genomes (Bucklin et al., 2018) still constitute an obstacle to conduct proper studies focusing on gene expression and selection in natural populations.

**Figure 1:**
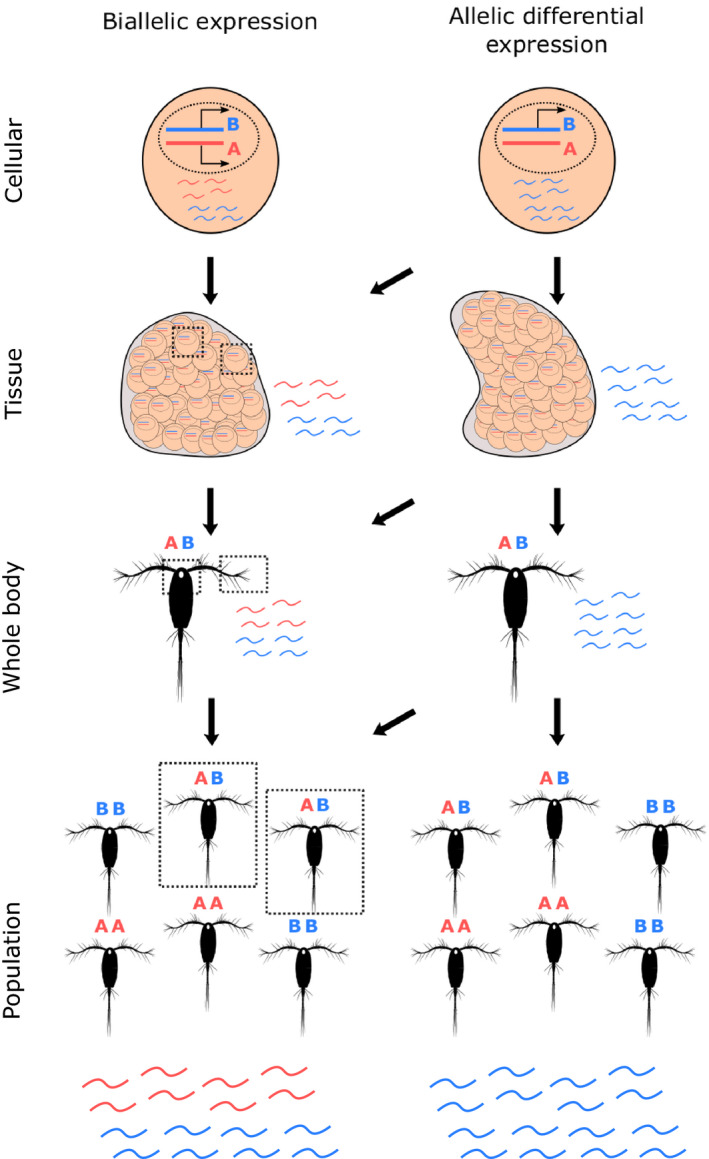
Allelic differential expression at population‐scale. (a) Different scales of allele‐specific expression detection for a heterozygous gene, from population to cellular levels. For a heterozygous genotype, ADE is understood as the difference in expression between two alleles of a single gene, opposed to strict biallelic expression. For clarity, the example of ADE presented here is monoallelic expression

In the present study, we proposed to measure the population‐scale allelic differential expression (psADE) of genes. psADE depends on the difference between alleles abundance at the genomic and transcriptomic level. At population scale, it aggregates differential expression between the two alleles at smaller scales (Figure 1). To measure psADE on small organisms, it would require the sequencing of several individuals at genomic and transcriptomic levels separately. An alternative approach could be to take advantage of the recent advances in metagenomic and metatranscriptomic sequencing of environmental samples, which offer a direct populational insight. In this context, polymorphic sites of a single species have to be extracted, allowing to evaluate whether the population‐scale relative expression of an allele deviates from its genomic frequency.

Copepods, and particularly species belonging to the *Oithona* genus, are small crustaceans forming the most abundant metazoan on Earth (Gallienne, 2001; Humes, 1994; Kiørboe, 2011). This abundance, reflecting strong adaptive capacities to environmental fluctuations, together with large hypothetic effective population size (Peijnenburg & Goetze, 2013; Riginos, Crandall, Liggins, Bongaerts, & Treml, 2016; Madoui et al. 2017; Arif et al. 2018) make this species suitable for population genomics analyses. In addition, they play an ecological key role in biogeochemical cycles and in the marine trophic food chain (Wassmann et al. 2006). In this study, we propose to detect psADE by focusing on the widespread epipelagic copepod, *Oithona similis* (Cyclopoida, Claus, 1866). We used environmental samples collected by the *Tara* Oceans expedition (Karsenti et al. 2011; Pesant et al. 2015) during its Arctic phase (2013) for which both metagenomic and metatranscriptomic data are available. Arctic Seas is an area where *O*.* similis* is known to be highly abundant (Blachowiak‐Samolyk, Kwasniewski, Hop, & Falk‐Petersen, 2008; Castellani et al., 2016; Dvoretsky, 2012; Zamora‐Terol, Nielsen, & Saiz, 2013). First, variants of *O*.* similis* were extracted and the genetic structure between the populations was studied. Then, we detected loci under psADE, under selection and under both psADE and selection. From these results, we tried to decipher the potential links between psADE, genomic differentiation and natural selection. Lastly, we investigated the molecular functions and biological processes of candidate loci under psADE and selection.

## 2 MATERIALS AND METHODS

### 2.1 Variant calling using Tara Oceans metagenomic and metatranscriptomic data

**Figure 2:**
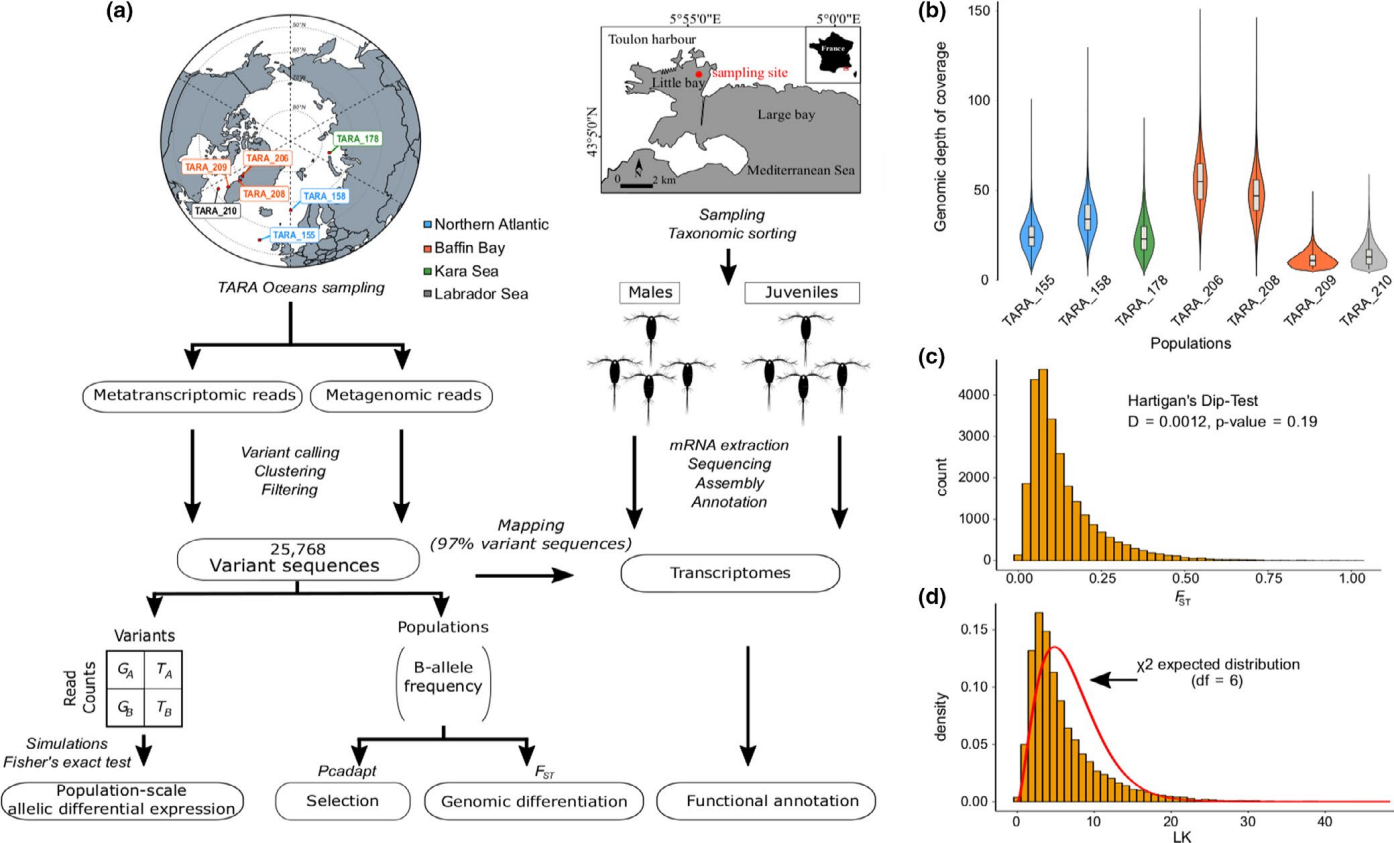
Genomic polymorphism data of *O*.* similis*. (a) Scheme representing the study, from samplings to analyses. (b) Genomic depth of coverage distributions of the set of 25,768 variants by sample. (c) *F*_ST_ distribution across the seven samples. (d) LK distribution. The red line represents chi‐squared theoretical distribution (*df* = 6)

We used metagenomic and metatranscriptomic reads generated from samples of the size fraction 20–180 µm collected in seven *Tara* Oceans stations (TARA_155, 158, 178, 206, 208, 209, and 210l Figure 2a) according to protocols fully described by Alberti et al. (2017) (Table S1). Because of the lack of a reference genome, the reference‐free variant caller *DiscoSNP++* (Uricaru et al. 2014; Peterlongo et al. 2017) was used to extract SNVs simultaneously from raw metagenomic and metatranscriptomic reads and was run using parameter –b 1. Only SNVs corresponding to biallelic loci with a minimum of 4x of depth of coverage in all stations were initially selected. Then, to capture loci belonging to *Oithona similis*, SNVs were clustered based on their loci co‐abundance across samples using density‐based clustering algorithm implemented in the R package dbscan (Ester, Kriegel, Sander, & Xu, 1996; Ram, Jalal, Jalal, & Kumar, 2010) and ran with parameters epsilon = 10 and minPts = 10. This generated three SNVs clusters, the largest of which contained 102,258 SNVs. To ensure only the presence of *O*.* similis* SNVs, we observed the fitting of the depth of coverage to the expected negative binomial distribution in each population (Figure S3). As the variant calling step is reference‐free, the alternative allele (here, B‐allele) is arbitrary set by *DiscoSNP++*. For each variant in each population, the B‐allele frequency (BAF) and the population‐level B‐allele relative expression (BARE) were computed as follows, $BAF=\frac{G_{B}}{G_{B}+G_{A}}$ and $BARE=\frac{T_{B}}{T_{B}+T_{A}}$, with *G_A_*, *G_B_* the metagenomic read counts supporting the reference and alternative alleles respectively and *T_A_*, *T_B_* the metatranscriptomic read counts supporting the reference and alternative alleles, respectively.

Biallelic loci were then filtered based on their metagenomic coverage. For each sample, the median and standard deviation σ of the distribution of metagenomic coverage of all biallelic loci were estimated. Biallelic loci must be characterized by a metagenomic coverage between a limit of median ± 2σ, with a minimum and maximum of 5× and 150× coverage in each sample to avoid low covered and multicopy genomic regions. To keep out rare alleles and potential calling errors, only variants characterized by a BAF comprised between 0.9 and 0.1, and a BARE between 0.95 and 0.05 in at least one population were chosen for the final dataset resulting in 25,768 biallelic loci (Table S5).

To ensure that these loci belong to *O*.* similis*, the global *F*‐statistics (or Wright's fixation index (Wright, 1951; Lewontin & Krakauer 1973) over the seven populations was computed as follows, *F*_ST_ = $\frac{\sigma^{2}}{\bar{p}\left(1-\bar{p}\right)}$, with $\bar{p}$ and σ^2^ being the mean allele frequency and the related variance, and its distribution was tested for unimodality via a Hartigans’ dip test (Hartigan & Hartigan, 1985). Moreover, LK statistics (Lewontin & Krakauer 1973) was computed as follows, LK = $\frac{n-1}{\bar{F_{\mathrm{ST}}}}F_{\mathrm{ST}}$ and compared with the expected chi‐squared distribution with *df* = n‐1, with n being the number of populations and $\bar{F_{\mathrm{ST}}}$ being the mean $F_{\mathrm{ST}}$ across all loci.

### 2.2 Population‐scale ADE detection using metagenomic and metatranscriptomic data

In each population, we first selected heterozygous loci variants (BAF ≠ {0,1}). We tested the correlation between BAF and BARE and modeled their relationship by a linear regression. Then, we computed *D = BAF‐BARE* and estimated the distribution parameters *µ* and *σ^2^* by fitting a normal distribution via *fitdist* function from *fitdistrplus* R package (Delignette‐Muller & Dutang, 2015).

We then tested the psADE of each variant using a two‐sided Fisher's exact test on a 2 × 2 table containing the read counts *G_A_*, *G_B_*, *T_A_*, and *T_B_*. Given the large number of tests, we applied the Benjamini and Hochberg *p*‐value correction (Benjamini & Hochberg, 1995) to control the False Discovery Rate (FDR). This generated seven sets of candidate loci under psADE, one set for each population.

### 2.3 Noise detection in population‐scale ADE using simulated data

To account for noise originating from potential sampling bias during sequencing, simulations were performed by generating sets of variants: (a) We modeled the distributions of the genomic depth of coverage of the loci (i.e., the sum of *G_A_* and *G_B_*) from each of the seven samples separately (Table S3) by a negative binomial (NB) distribution (Robinson & Smyth, 2007) and estimated seven *µ* and *θ* (the NB mean and shape parameters); (b) The relationship among the seven samples between the observed *µ* and *θ* by a linear regression (Figure S4), allowing us to estimate a shape parameter *θ* for any given mean *µ*; (c) A‐allele frequencies followed a U‐shaped distribution, approximated by a beta distribution of shape parameters *α* and *β*; (d) The expression level (i.e., the sum of *T_A_* and *T_B_*) was modeled by a gamma distribution of shape and rate parameters *a* and *b*. These estimations were performed with *fitdist* function of *fitdistrplus* R package (Delignette‐Muller & Dutang, 2015) and are presented in Figure S3.

To simulate the genomic A‐allele frequency for one biallelic loci in a given population, we extracted one random deviates from its beta distribution of shape parameters *α*, *β* estimated previously for each population in (c). The A‐allele frequency was multiplied by the estimated *µ* to obtain an expected genomic read count of allele A, $\bar{G_{A}}$. In the same way, $\bar{G_{B}}$ was obtained by the difference between *µ_n_* and $\bar{G_{A}}$. Simulated genomic read counts for allele A and B were then obtained by generating a random value from negative binomial distributions of parameters *µ_A_* = $\bar{G_{A}}$ and *µ_B_* = $\bar{G_{B}}$ respectively, and the corresponding size parameters *θ_A_* and *θ_B_* estimated using the linear regression between *θ* and *µ* as described above in (b).

A locus expression level was then computed by generating random deviates following a gamma distribution of shape *a* and rate *b* estimated previously for each population in (d). Under the null hypothesis, the allele abundance at genomic and transcriptomic level is the same (see Table S3), the expected transcriptomic read count of allele A, $\bar{T_{A}}$, was generated by multiplying the genomic A‐allele frequency previously computed and the locus expression level. In the same way, $\bar{T_{B}}$ was obtained by the difference between the locus level expression and $\bar{T_{A}}$. Finally, simulated transcriptomic read count *T_A_* and *T_B_* were obtained by generated random values from a Poisson distribution of parameter *λ_A_* = $\bar{T_{A}}$ and *λ_B_* = $\bar{T_{B}}$, respectively. All simulations were performed using *lm*, *rbeta*, *rnbinom*, *rgamma,* and *rpois* R functions.

To formally test for psADE due to noise (i.e., under the null hypothesis), seven sets of 50,000 loci were simulated using the seven sets of parameters learnt for each sample. Fisher's exact test was performed only on heterozygous loci with a non‐null expression level, and simulated variants with a *q*‐value < 0.05 (Benjamini‐Hochberg correction) were considered as noisy psADE, as described above.

### 2.4 Estimation of genomic differentiation and detection of variants under selection

Pairwise‐*F*_ST_ was computed as follows, *F*_ST_ = $\frac{\sigma_{i}^{2}}{{\bar{p}}_{i}\left(1-{\bar{p}}_{i}\right)}$, for each locus between each pair of populations *i* and the median pairwise‐*F*_ST_ was retained to measure the genomic differentiation between each population. A Mantel test was performed to test for isolation‐by‐distance between median pairwise‐*F*_ST_ and geographic Euclidean distances using *vegan* v2.5‐2 (Oksanen et al. 2018) and *geosphere* v1.5–7 (Hijmans, 2017) R packages. The *pcadapt* R package v4.0.2 (Luu, Bazin, & Blum, 2017) was used to detect selection among populations from the B‐allele frequency matrix. The computation was run on “*Pool‐seq*” mode, with a minimum allele frequency of 0.05 across the populations, and variants with a corrected Benjamini and Hochberg *p*‐value < .05 were considered under selection.

### 2.5 Modeling psADE with population differentiation

The seven sets of candidate loci under psADE were crossed to identify variants under psADE in several populations (named “shared psADEs”) and all nonempty, nonoverlapping crossings between populations were represented by an upset plot.

To test whether populations characterized by weak genetic differentiation tend to share more loci under psADE than genetically distant populations, we modeled the number of shared psADEs between populations by genomic differentiation using a nonlinear model: *y = a e^‐bx^ + c*, with y being the number of shared ADE and x the genomic differentiation. The latter was estimated by computing the median *F*_ST_ of each nonempty crossing set of populations as described above, with *i* being here the considered set of populations. To find the starting values, the model was linearized as follows, *log(y‐c_0_) ≈ log(a) + bx*, with c_0_ = min(y)*0.*5* and *a* and *b* parameters were estimated via the *lm* R function. The nonlinear model was then applied, and least squares estimates were used via the *nls* R function. Pearson's correlation between the fitted and empirical values was then computed via the *cor*.*test* R function.

### 2.6 psADE and link with natural selection

To identify alleles under both psADE and natural selection, the set of variants under psADE in each population was crossed with the set of loci detected under selection. The size of the intersection was tested by a hypergeometric test, *H*(*q*,*m*,*n*,*k*), with *q* being number of alleles under psADE in the population and under selection (size of intersection), *m* being the total number of alleles under selection, *n* being the total number of variants under neutral evolution, and *k* being the total number of alleles under psADE in the tested population. We considered that, in a given population, the number of alleles under both psADE and selection was significantly higher than expected by chance for *p*‐value < .05.

### 2.7 ***Material sampling, mRNA extraction, and Mediterranean O*** .*** similis transcriptomes sequencing***

To conduct a functional analysis, Mediterranean *O*.* similis* transcriptomes were produced. *Oithona similis* specimens were sampled at the North of the Large Bay of Toulon, France (Lat 43°06’ 02.3” N and Long 05°56’ 53.4” E). Sampling took place in November 2016. The samples were collected from the upper water layers (0–10 m) using zooplankton nets with a mesh of 90µm and 200 µm (0.5 m diameter and 2.5 m length). Samples were preserved in 70% ethanol and stored at −4°C. From the Large Bay of Toulon samples, *O*.* similis* individuals were isolated under the stereomicroscope (Nishida, 1985; Rose, 1933). We selected two different development stages: four copepodites (juveniles) and four adult males. Each individual was transferred separately and crushed, with a tissue grinder (Axygen) into a 1.5 ml tube (Eppendorf). Total mRNAs were extracted using the ‘RNA isolation’ protocol from NucleoSpin RNA XS kit (Macherey‐Nagel) and quantified on a Qubit 2.0 with a RNA HS Assay kit (Invitrogen) and on a Bioanalyzer 2100 with a RNA 6000 Pico Assay kit (Agilent). cDNA was constructed using the SMARTer‐Seq v4 Ultra low Input RNA kit (ClonTech). The libraries were built using the NEBNext Ultra II kit for paired‐end sequencing with an Illumina HiSeq2500. After adaptors trimming, only reads with a mean Phred score > 20 were kept.

### 2.8 Transcriptomes assembly and annotation

Each read set was assembled with Trinity v2.5.1 (Haas et al. 2013) using default parameters and transcripts were clustered using cd‐hit v4.6.6 (Fu, Niu, Zhu, Wu, & Li, 2012) using ‐c 0.9 ‐aS 0.8 ‐aL 0.8 parameters (Table S2). To ensure the classification of the sampled individuals, each ribosomal read set were detected with SortMeRNA (Kopylova, Noé, & Touzet, 2012) and mapped with bwa v0.7.15 using default parameters (Li & Durbin, 2009) to 82 ribosomal 28S sequences of *Oithona* species used in Cornils, Wend‐Heckmann, & Held, 2017 (Figure S2). The transcriptome assemblies were annotated with Transdecoder v5.1.0 (Haas et al. 2013) using default settings to predict the open reading frames (ORFs) and protein sequences (Table S2). In parallel, homology searches were also included as ORF retention criteria for Transdecoder; the peptide sequences of the longest ORFs were aligned on *Oithona nana* proteome (Madoui et al. 2017) using DIAMOND v0.9.22 (Buchfink, Xie, & Huson, 2014). Protein domain annotation was performed on the final ORF predictions with Interproscan v5.17.56.0 (Jones et al. 2014) and a threshold of *E*‐value < 10^–5^ was applied for Pfam annotations. Finally, homology searches of the predicted proteins were done against the nonredundant NCBI protein database, restricted to Arthropoda (taxid: 6656), with DIAMOND v0.9.22.

### 2.9 Variant functional annotation

The variant functional annotation was conducted in two steps. First, the variant sequences were mapped on the previously annotated *O*.* similis* transcripts using the “VCF_creator.sh” program of *DiscoSNP++*. Secondly, a variant annotation was carried out with SNPeff (Cingolani et al. 2012) to identify the location of variants within transcripts (i.e., exon or UTR) and to estimate their effect on the proteins (missense, synonymous or nonsense). The excess of candidate variant annotations was tested for the different classes of SNPEff, for the three sets of variants under selection, psADE, and both, by comparing to the total sets of annotated variants. A significant excess was considered for a hypergeometric test *q*‐value < 0.05, after Benjamini‐Hochberg correction.

### 2.10 Gene enrichment analysis

To identify putative biological function or processes associated to the variants, a domain‐based analysis was conducted. The Pfam annotation of the transcripts carrying variants categorized as psADE and selection was used and compared to Pfam annotation of the total sets of variants with a hypergeometric test for enrichment. A significant excess was declared for a *q*‐value < 0.05 (Benjamini‐Hochberg correction). To complete the domain‐based analysis, the functional annotations obtained from the homology searches against the nr were manually curated.

## 3 RESULTS

### 3.1 Extracting polar Oithona similis variants from environmental samples

From metagenomic and metatranscriptomic raw data of seven sampling stations (Figure 2a), we identified 102,258 variants using a reference‐free approach. Among them, 25,768 expressed *O*.* similis* variants were retrieved after filtering. To ensure that the variants belonged to *O*.* similis*, we performed three different analyses. First, in each sample, the distributions of variable loci depth of coverage were unimodal (Figure 2b) and fitted the expected negative binomial distributions (Figure S3). Secondly, 97% of 25,768 variants were mapped on Mediterranean *O*.* similis* transcriptomes (Figure 2a). Third, the global distribution of *F*_ST_ of the seven populations was unimodal (Hartigans’ dip test, D = 0.0012, *p*‐value = .19) with a low median *F*_ST_ at 0.1 (Figure 2c), confirmed by the pairwise‐*F*_ST_ distributions (Figure S6d). Finally, the LK distribution over all the loci followed the expected chi‐squared distribution (Figure 2d), showing that most of the loci follow the neutral evolution model, as expected in a single species.

### 3.2 Oithona similis genomic differentiation in Arctic Seas

**Figure 3:**
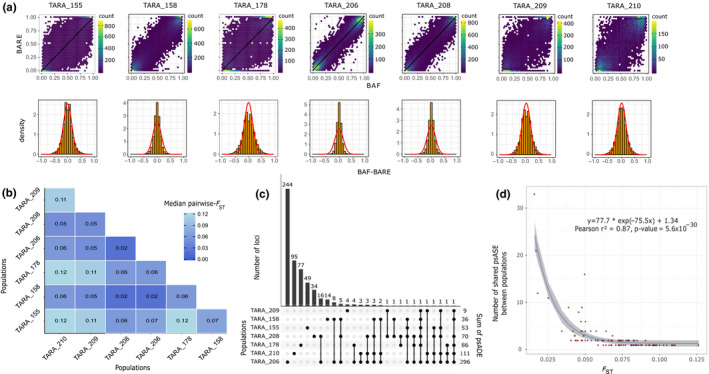
Population‐scale allelic differential expression and its link with genomic differentiation. a, Each column corresponds to a population. Upper panel represents the relation between BAF and BARE, each hexagone corresponds to an area containing the number of variants indicated by the color scale. Black lines are the linear regression curves. Lower panel represents the distribution of BAF‐BARE. The red lines correspond to the Gaussian distribution estimated from the data. b, Pairwise *F*_ST_ matrix. The median (mean) of each pairwise‐*F*_ST_ distribution computed on allele frequencies is indicated c, Upset plot of psADE detection in the seven populations. Each bar of the upper plot corresponds to the number of variants under psADE in the combination of population(s) indicated by black dots in the lower plot. d, Genomic differentiation and shared psADE. Each dot is a combination of population as presented in the lower panel of the upset plot. The blue line represents the nonlinear regression curve estimated from the data and 95% confidence interval in gray

The seven populations were globally characterized by a weak to moderate differentiation, with a maximum median pairwise‐*F*_ST_ of 0.12 between populations from TARA_210 and 155/178 (Figure 3b, Figure S6d). Populations from stations TARA_158 (Norway Current), 206 and 208 (Baffin Bay) were genetically closely related, with the lowest median pairwise‐*F*_ST_ (0.02), despite TARA_158 did not co‐geolocalize with the two other stations. The four other populations (TARA_155, 178, 209, and 210) were equally distant from each other (0.1–0.12). Finally, TARA_158, 206, and 208 on one side and TARA_155, 178, 210, and 209 on the other side showed the same pattern of differentiation (0.05–0.07). A Mantel test was performed and revealed no correlation between *F*_ST_ and geographic distances (*r* = 0.34, *p*‐value = .13; Figure S7).

### 3.3 Detection of population‐scale ADE

As expected, most of the loci presented a strong correlation between B‐allele frequency and B‐allele relative expression (Figure 3a, Table S3). Thus, we observed the difference between BAF and BARE, which followed a Gaussian distribution centered on 0 in each population (Figure 3a, Table S3). The number of SNVs tested for psADE varied between 13,454 and 22,578 for TARA_210 and 206 respectively. We found a significant amount of variants under psADE in each population under a Fisher's exact test (Figure 3c, Table S4). Potential noise due to sample bias during sequencing was estimated by simulations for each of the seven population and was relatively negligible compared with real data. Distributions of simulated *p*‐values under null hypothesis (i.e., no psADE) and *O*.* similis* empirical *p*‐values (Figure S5) show higher amount of significant *O*.* similis* *p*‐values compared with the simulated ones, resulting in a proportion of true‐positives psADEs varying from 70% to 100% (Table S4).

Overall, we found 572 variants under psADE, including 513 population‐specific psADEs, and 59 psADEs shared by several populations (Figure 3c). Remarkably, 29 psADEs out of the 59 were present only in the populations from TARA_158, 206, and 208 that correspond to the genetically closest populations, leading us to compare the relationship between sharing psADEs and population differentiation. By comparing the number of shared psADEs in the different sets of populations to their genomic differentiation, we found a negative trend between the two (with a strong negative exponential slope estimate), illustrated by a significant correlation between nonlinear fitted and empirical values (0.87, *p*‐value 5.6 × 10^−30^, Figure 3d). This modeling shows that genetically close populations tend to share more variants under psADE.

### 3.4 Loci under population‐level ADE and selection in Arctic populations

The set of variants was tested for selection using *pcadapt*. The PCA decomposed the genomic variability in six components (Figure S6a–c); the first two components discriminated TARA_155 and 178 from the others (32% and 28.1% variance explained, respectively), and the third component differentiated TARA_210 and 209 (19.5%). The fourth principal component separated TARA_209 and 210 from 158/206/208 (11.3%), with the last two concerning TARA_158/206/208. Globally, these results dovetailed with the *F*_ST_ analysis, with details discussed later. Finally, we detected 674 variants under selection, representing 2.6% of the dataset (corrected *p*‐value < .05).

**Figure 4:**
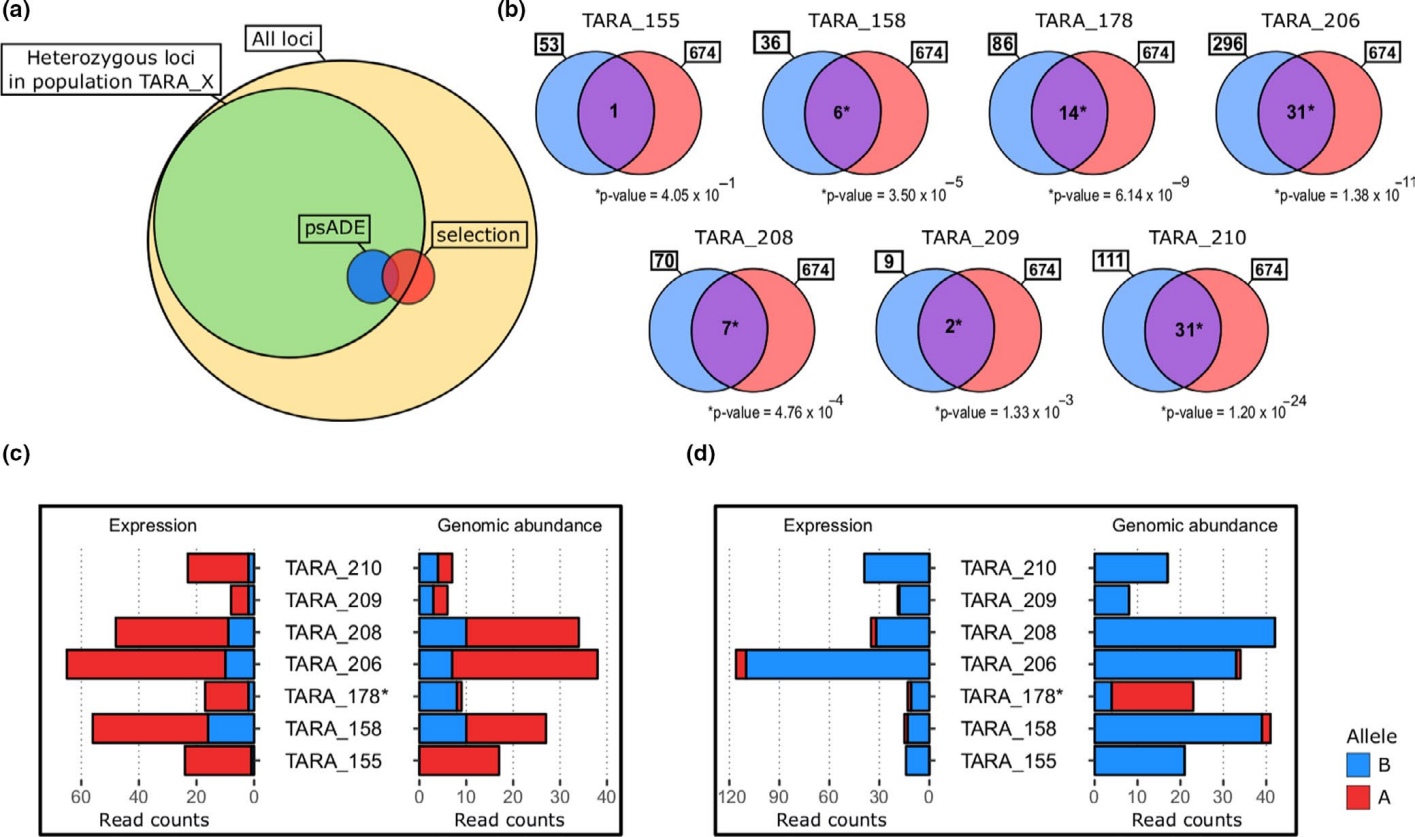
Crossing psADE and selection. (a) Scheme representing the method; in yellow the total dataset of SNVs, in green the tested SNVs in one population, in blue SNVs under psADE in this population, in red the SNVs targeted by selection. (b) Crossing the candidate variants under psADE (blue) and those under selection (red) for each population. The hypergeometric *p*‐value corresponds to the significance of the amount of variants both under psADE in the considered population and under selection (purple). (c, d) Genomic abundance and expression profiles of variants 20,286,969 and 1,522,691. The first one is under psADE in TARA_178 for allele A (*), which is fixed in TARA_155. The second one is under psADE in favor of allele B in TARA_178 (*) and reaching near fixation for the same allele in all the other populations

The seven sets of variants under psADE were crossed with the set of variants under selection (Figure 4a). The size of the intersections ranged from 1 to 31 variants (TARA_155 and 206/210) and was significantly higher than expected by chance for all the populations but TARA_155 (Figure 4b, hypergeometric test *p*‐value < .05). It represented a total of 84 unique variants under selection and psADE in at least one population, corresponding to 15% and 12% of variants under psADE and under selection, respectively. Two main different genomic and expression profiles can be observed (Figure S8). First, as illustrated in Figure 4c, loci can show an allele under psADE but not fixed in one population (here allele A in TARA_178) and fixed in another (TARA_155). A second observed pattern, more extreme, concerns variants as exampled in Figure 4d, which presents an allele favored by psADE (allele B in TARA_178), with a low genomic frequency and fixed in nearly all the other populations.

### 3.5 Functional analysis of transcripts under population‐scale ADE and selection

The full dataset of variants was positioned on the eight transcriptomes to extract putative functional information, with a total of 25,048 variants (97% of total) successfully mapped on 16,272 transcripts. First, SNPEff was used to estimate the localization of variants inside the transcripts, and an enrichment was estimated for all categories, and for three sets of variants categorized respectively as under selection, psADE and both (Figure S9). Overall, the two first sets showed a significant excess of variants in 3’ UTR. Plus, among the variants under psADE, one was categorized as a “stop gained” and one as a “stop retained”. However, variants under psADE and selection did not show any excess of specific effect.

Among the 84 loci identified under psADE and selection, 80 were located on *O*.* similis* transcripts (Table S6). Amid these transcripts, 64 (76%) were linked to at least one Pfam domain (61 different domains) and 59 (70%) to a functional annotation from the nr database. From this total of 61 Pfam domains, 23 presented a significant excess compared with domains present in the global set of transcripts, corresponding to 21 transcripts (Figure S10). On the latter, three transcripts were involved in nervous system features: omega‐amidase NIT2, vang‐like 2B protein, and 5‐oxoprolinase.

## 4 DISCUSSION

### 4.1 ***Genomic and transcriptomic variation data belong to a single*** *Oithona* ***similis lineage***

Because genomes of small animals like copepods are difficult to reconstruct, we used *DiscoSNP++*, a reference‐free variant caller to extract variants from metagenomic data, that already showed its accuracy on *Tara* Oceans metagenomic data (Arif et al. 2018).

Global populations of *O*.* similis* are known to be composed of cryptic lineages across oceanic basins (Cornils et al., 2017). It is also known that this species in highly abundant among other copepods in Arctic Seas (Blachowiak‐Samolyk et al., 2008; Castellani et al., 2016; Dvoretsky, 2012; Zamora‐Terol et al., 2013). Thus, the assessment that the extracted variants from the seven samples used in our study belongs to the same *O*.* similis* cryptic lineage was a prerequisite for further analyses. Three different analyses support this assumption. First, the distribution of depth of coverage in each of the seven samples followed the expected negative binomial distribution (Supplementary Figure S3). Indeed, the possibility to observe these patterns in the presence of different species would require them to be equally co‐abundant, which is unlikely. Thus, this covariation of the depth of coverage of these variants supports the single species genome origin. Secondly, the high proportion of variants (97%) mapped on the Mediterranean *O*.* similis* transcriptomes, another cryptic lineage (Cornils et al., 2017), showed that the variant clustering method was efficient to regroup loci of *O*.* similis*. Finally, the unimodal distribution of *F*_ST_ showed that these populations of *O*.* similis* belong to the same polar cryptic species, and that most of the loci are under neutral evolution. Altogether, these results show that we were able to retrieve polymorphic data of a single species, *O*.* similis*, on which population differentiation analyses and psADE detection can be undertaken.

### 4.2 Oithona similis populations are weakly differentiated within the Arctic Seas

We observed that the seven populations examined showed low genomic differentiation, despite the large distances separating them, which was illustrated by a nonsignificant Mantel test for isolation‐by‐distance (Figure S7). *F*_ST_ and *pcadapt* analyses both showed the same patterns of genomic differentiation. First, the differentiation of populations from TARA_155 and 178 is relatively high compared with the others. Secondly, the geographically close populations from TARA_210 and 209 present a relatively high differentiation (median pairwise‐*F*_ST_ of 0.11, PC3). This could be explained by the West Greenland current acting as a physical barrier between the populations, which could lead to reduced gene flow (Myers, Donnelly, & Ribergaard, 2008). At last, the strong link between TARA_158 from Northern Atlantic current and TARA_206/208 from the Baffin Bay is the most intriguing. Despite the large distances that separate the first one from the others, these three populations are well connected. Based on this weak structure and that most of loci follows a neutral evolution (Figure 2d), outliers detected by *pcadapt* probably are truly under selection and not due to specific population differentiation.

Metagenomic data enable to draw the silhouette of the gene flow between populations but lacks resolution when dealing with intrapopulation structure. However, our findings are concordant with previous studies underpinning the large‐scale dispersal, interconnectivity of marine zooplankton populations in other oceans, at diverse degrees (Blanco‐Bercial & Bucklin, 2016; Goetze, 2005; Höring, Cornils, Auel, Bode, & Held, 2017; Peijnenburg & Goetze, 2013). Weak genetic structure in the polar region was highlighted for other major Arctic copepods like *Calanus glacialis* (Weydmann, Coelho, Serrão, Burzyński, & Pearson, 2016) and *Pseudocalanus* species (Aarbakke, Bucklin, Halsband, & Norrbin, 2014). The absence of structure was explained by ancient diminutions of effective population size due to past glaciations (Aarbakke et al., 2014; Bucklin & Wiebe, 1998; Edmands, 2001), or high dispersal and connectivity between the present‐day populations due to marine currents (Weydmann et al., 2016). Using Lagrangian travel time or dispersal probabilities could help to estimate how much marine currents explain this observed genomic differentiation.

### 4.3 *Population‐scale ADE in O*.* similis populations and its link with differentiation and selection*

We were able to detect variants under psADE in the seven populations. First, allele frequency and relative expression are strongly correlated in the data, showing as expected that the more an allele is observed at the genomic level, the more this allele is expressed. Simulations performed showed that although this sequencing bias noise is present in our data, it does not significantly affect psADE detection. Among the variants under psADE, a large part was population‐specific and a minority was under psADE in several populations. From the latter, we showed that closely related populations tended to share more variants under psADE than other more differentiated populations, meaning that a genetic basis partially shapes these psADEs. This result shows that psADE allows the observation of similar allelic expression variation in individuals sampled from geographically very distant populations (>4,000 km for TARA_206 and 158) but having very strong genetic relatedness. This finding clearly suggests that these variations of expression are probably controlled by the same regulatory genetic drivers.

We estimated the number of loci under psADE that were also targeted by natural selection. A significant amount of SNVs (84) was subject to selection among the seven populations and to psADE in at least one population, meaning that in Arctic populations of *O*.* similis*, psADE and natural selection affect the same genomic regions.

Two main patterns can be observed in the candidate loci. The first one (as in Figure 4c) shows one allele under psADE and selection in different populations (here allele A in TARA_178 and TARA_155 respectively). A second observed pattern concerns loci as exampled in Figure 4d (see also Figure S8), where an allele (here B) is nearly fixed in six populations and under psADE in one population (here TARA_178), with this psADE due to a low abundance and high expression. In this population, the allele B sees its frequency decreasing because another allele appears in this population. However, since the latter is under‐expressed, it could mean that it is a deleterious mutation, and strong regulatory elements or molecular mechanisms repress its expression, or that even a small expression enables a higher fitness for individuals carrying it, or that the allele favored by psADE is the one enabling higher fitness, leading to fixation in other populations. Ultimately, although determining how psADE and selection interact remains beyond the scope of this study, we can hypothesize from these observations that the action of the two mechanisms on a locus can be (a) independent, psADE and selection acting separately, (b) sequential, with psADE acting before, while or after selection occurs.

The process of acclimation through gene expression and the link with genetic variation and adaptation have been studied widely in several organisms (Fay & Wittkopp, 2008; Signor & Nuzhdin, 2018; Williams, Chan, Cowley, & Little, 2007). In a first study in human, a link has been established between gene expression and selection, affecting particular genes and phenotypes, looking at *cis*‐acting SNPs (Fraser, 2013). In a second study, the team was able to detect loci under ADE and selection at the same time in different human populations (Tian et al. 2018). Also, approaches in a plant model, *Capsella grandiflora*, a species characterized by weak population structure and large effective population size, emphasized the relative impact of purifying selection and positive selection on *cis*‐regulatory variation in populations (Josephs, Lee, Stinchcombe, & Wright, 2015; Steige, Laenen, Reimegård, Scofield, & Slotte, 2017). Our study, by focusing on whole populations of numerous individuals, offers further clues to understand the link between gene expression variation and selection.

Further investigations including replicates, more populations, and the production of a genome and genotypes would help to confirm our results, disentangle the different causes of psADE, and question the link between psADE and selection.

### 4.4 Functional insights of natural selection and population‐scale ADE in Oithona similis

By analyzing the functional annotation of the variants with SNPEff, we found a significant excess of variants under selection and variants under psADE located in 3’UTR, but no excess in variants under selection and psADE. Variations in these regions are known to both affect transcription efficiency through mRNA secondary structures, stability, and location (Matoulkova, Michalova, Vojtesek, & Hrstka, 2012; Mignone, Gissi, Liuni, & Pesole, 2002), leading to affect the function of the gene. However, no clear pattern was observed among the candidate variants.

On the 674 loci under selection, some variants were located in transcripts annotated by homology search (Table S6) as pantothenate kinase, glycine receptors/GABA receptors, and FMRFamide receptor. The same genes are also under selection in *Oithona nana* populations of Mediterranean (Madoui et al. 2017), stressing their evolutive importance. To date, variants located in transcripts linked to FMRFamide, glycine, and GABA receptors are also found among the 572 variants under psADE, but not in the 84 candidate variants under psADE and selection.

Glutamate, GABA and glycine are known neurotransmitters in arthropods motor neurons (Smarandache‐Wellmann, 2016). Pantothenate kinase, an enzyme catalyzing the phosphorylation of vitamin B5, constituting the first step in Coenzyme‐A biosynthesis pathway, is linked to neurodegenerative diseases in human and *Drosophila* (Pandey, Turm, Bekenstein, Shifman, & Kadener, 2013). Among the transcripts under psADE and selection, three are of specific interests, as they are also involved in nervous system. Omega‐amidase NIT2 is an enzyme that produces α‐ketoglutaramate, a precursor of glutamate and GABA. The 5‐oxoprolinase produces glutamate from 5‐oxoproline. Finally, vang‐like protein 2B is involved in the formation of ommatidies in *Drosophila* (Leung et al. 2016). Other studies focusing on axon myelination in calanoid species illustrate how nervous system can play an important role in copepod evolution (Lenz, 2012; Weatherby, Davis, Hartline, & Lenz, 2000). From our results, more functional analyses would allow a better characterization of these genes, but it reveals the potential evolutionary importance of nervous system in copepods.

## 5 CONCLUSION

Gene expression variation is thought to play a crucial role in acclimation and adaptive history of natural populations. Herein, we integrated metagenomic and metatranscriptomic data to detect ADE at the population level in populations of copepods. Then, we demonstrated the link between psADE and population differentiation on one hand and with natural selection on the other hand, by providing a quantitative observation of this phenomenon and its impact on specific biological features of copepods. In the future, we will try to expand these observations to other organisms and question the nature of the link between psADE and natural selection.

## CONFLICT OF INTEREST

The authors declare no competing interests.

## AUTHOR CONTRIBUTION

**Romuald Laso‐Jadart:** Conceptualization (equal); Formal analysis (lead); Investigation (lead); Methodology (equal); Writing‐original draft (lead). **Kevin Sugier:** Resources (lead). **Emmanuelle Petit:** Resources (lead). **Karine Labadie:** Resources (lead). **Pierre Peterlongo:** Software (supporting); Writing‐review & editing (supporting). **Christophe Ambroise:** Methodology (supporting); Validation (supporting); Writing‐review & editing (equal). **Patrick Wincker:** Funding acquisition (lead); Supervision (supporting); Writing‐review & editing (supporting). **Jean‐Louis Jamet:** Resources (lead); Writing‐review & editing (supporting). **Mohammed‐Amin Madoui:** Conceptualization (lead); Formal analysis (supporting); Investigation (equal); Methodology (equal); Supervision (lead); Validation (equal); Writing‐original draft (supporting); Writing‐review & editing (lead).

## Supporting information

Supplementary Material

Table S5

Table S6

## Data Availability

All data are available at ENA (European Nucleotide Archive); see Table S1.
