# Supplementary material for: Investigating population‐scale allelic differential expression in wild populations of Oithona similis (Cyclopoida, Claus, 1866)
Source: Ecol Evol. 2020 Aug 4;10(16):8894–905. doi: 10.1002/ece3.6588 (PMC7452778; doi:10.1002/ece3.6588)
Supplement: Supplementary file 1 — Supplementary Material [file ECE3-10-8894-s001.doc]

Investigating Population-scale Allelic Differential Expression in Wild Populations of *Oithona similis* (Cyclopoida, Claus 1866)

Romuald Laso-Jadart^1,6*^, Kevin Sugier^1^, Emmanuelle Petit^2^, Karine Labadie^2^, Pierre Peterlongo^3^, Christophe Ambroise^4^, Patrick Wincker^1,6^, Jean-Louis Jamet^5^, Mohammed-Amin Madoui^1,6*^

^1^Génomique Métabolique, Genoscope, Institut François Jacob, CEA, CNRS, Univ Evry, Université Paris-Saclay, Evry, France.

^2^CEA, Genoscope, Institut de Biologie François Jacob, Université Paris-Saclay, Evry,

91057, France.

^3^Univ Rennes, CNRS, Inria, IRISA - UMR 6074, F-35000 Rennes.

^4^LaMME, CNRS, Univ Evry, Université Paris-Saclay, Evry, France

^5^Université de Toulon, Aix-Marseille Université, CNRS/INSU/IRD, Mediterranean Institute of Oceanology MIO UMR 110, CS 60584, 83041 Toulon cedex 9, France.

^6^Research Federation for the study of Global Ocean Systems Ecology and Evolution, FR2022/Tara Oceans GO-SEE, 3 rue Michel-Ange, 75016 Paris, France

^*^Corresponding authors. Emails: [rlasojad@genoscope.cns.fr](mailto:rlasojad@genoscope.cns.fr %26) & [amadoui@genoscope.cns.fr](mailto:amadoui@genoscope.cns.fr)

**Supplementary Figures & Tables**

[Supplementary Figure 1 : General pipeline 3](#__RefHeading___Toc41992056)

[Supplementary Figure 2 : Validation of taxonomic assignation. 4](#__RefHeading___Toc41992057)

[Supplementary Figure 3 : *Oithona similis* depth of coverage, allele frequency and expression level of biallelic loci in seven *Tara* Oceans samples. 5](#__RefHeading___Toc41992058)

[Supplementary Figure 4 : Linear regression between negative binomial distribution parameters *µ* and *θ*. 9](#__RefHeading___Toc41992059)

[Supplementary Figure 5 : Distributions of p-values from psADE detection performed on simulated and empirical data. 10](#__RefHeading___Toc41992060)

[Supplementary Figure 6 : Population genomic differentiation. 12](#__RefHeading___Toc41992061)

[Supplementary Figure 7 : Genomic differentiation and geographic distance. 13](#__RefHeading___Toc41992062)

[Supplementary Figure 8 : Metagenomic and metatranscriptomic profiles of candidate loci. 14](#__RefHeading___Toc41992063)

[Supplementary Figure 9 : Functional localization and effect of variants by SNPEff 18](#__RefHeading___Toc41992064)

[Supplementary Figure 10 : Pfam enrichment in candidate loci 19](#__RefHeading___Toc41992065)

[Supplementary Table S1 : *Tara* Oceans samples and *Oithona similis* Mediterranean transcriptomes accession numbers 20](#__RefHeading___Toc41992066)

[Supplementary Table S2 : *Oithona similis* Mediterranean transcriptomes summary. 21](#__RefHeading___Toc41992067)

[Supplementary Table S3 : Distribution parameters of genomic depth of coverage, variant frequency and expression level. 22](#__RefHeading___Toc41992068)

[Supplementary Table S4 : Estimation of false-positives in psADE detection. 23](#__RefHeading___Toc41992069)

# Supplementary Figure 1 : General pipeline


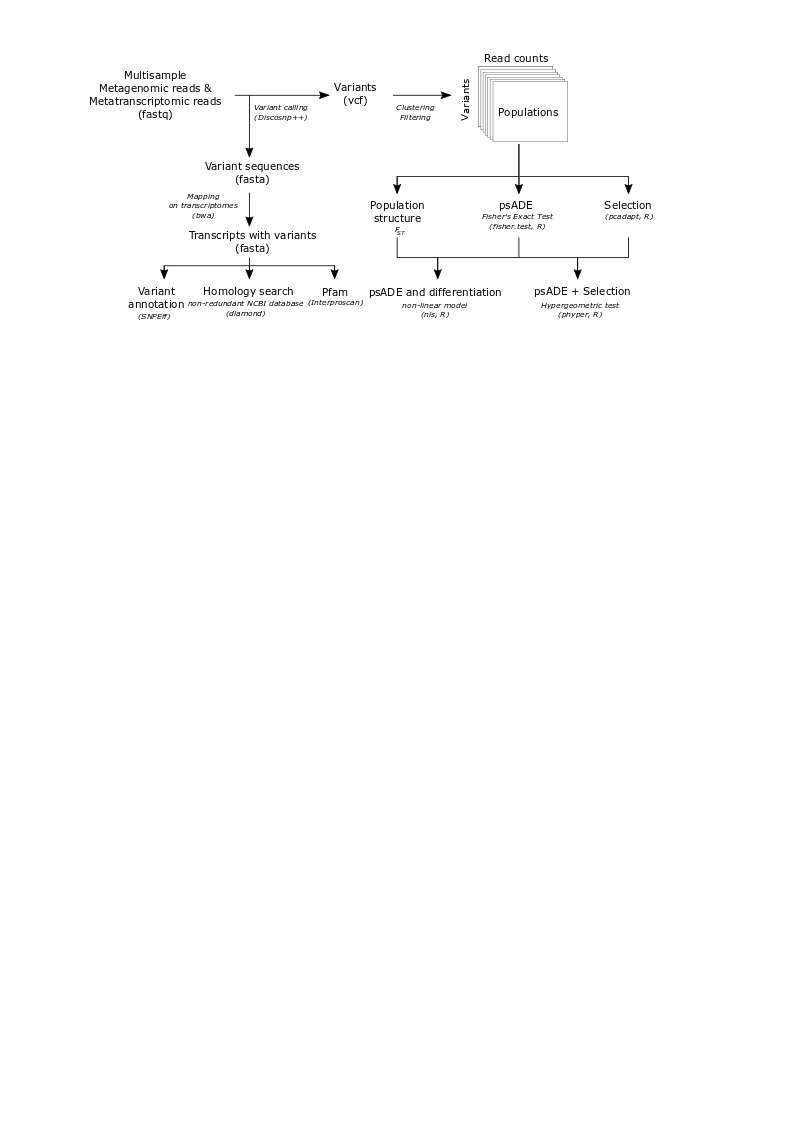


# Supplementary Figure 2 : Validation of taxonomic assignation.

In rows are represented the 82 accession numbers of *Oithona* species 28S sequences. In bold, type localities of *O. similis* as described in Cornils et al., 2017. In columns are represented ribosomal read sets of the eight individuals.


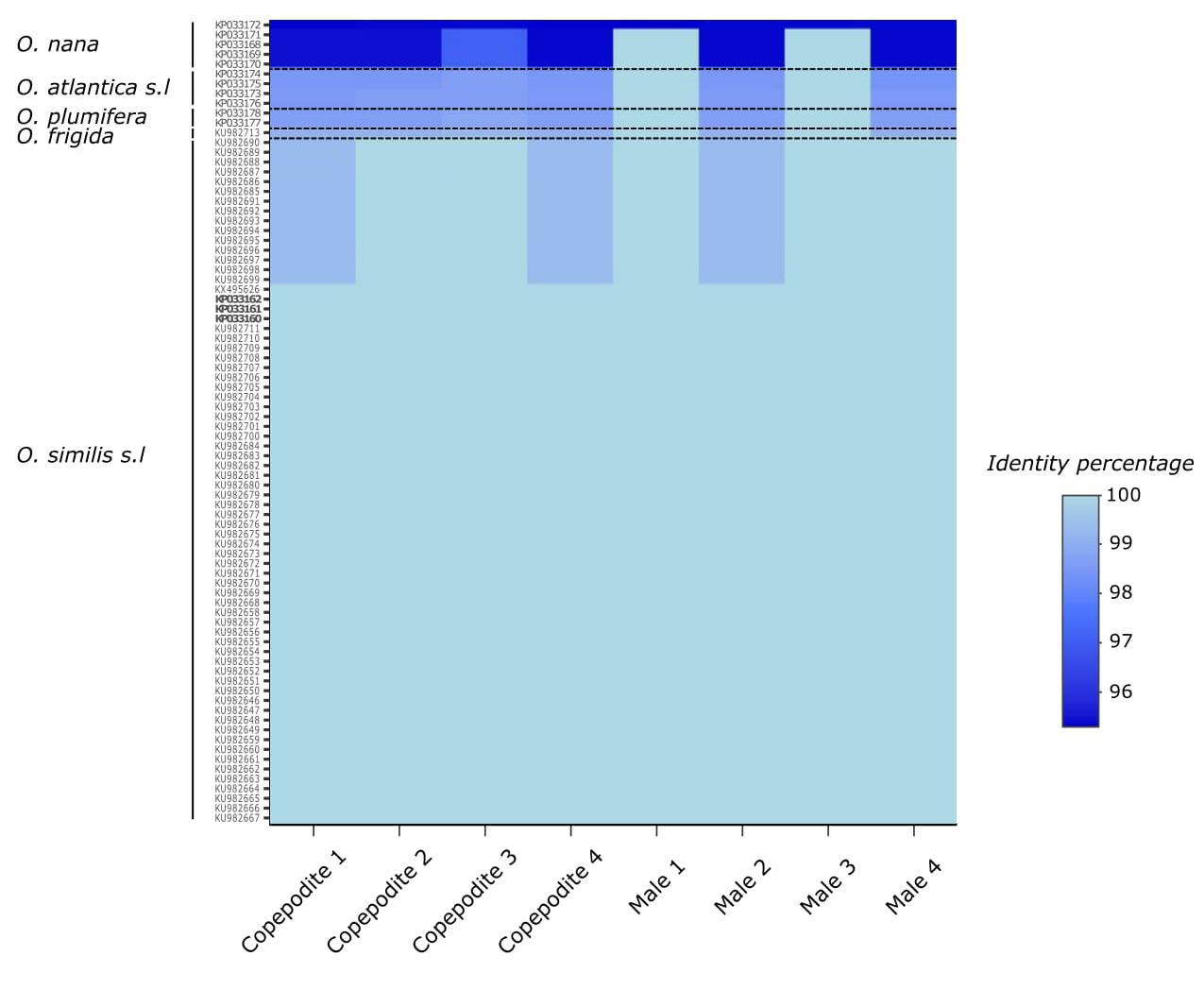


# Supplementary Figure 3 : *Oithona similis* depth of coverage, allele frequency and expression level of biallelic loci in seven *Tara* Oceans samples.

From a to g : TARA_155, 158, 178, 206, 208, 209, 210. For each population, are shown three fits from up to bottom : depth of coverage fitting a negative binomial distribution ; allele frequencies fitting a beta distribution ; expression levels fitting a gamme distributions. In red are represented the theoretical estimates. In black are represented the observed values.

**a**

**b**


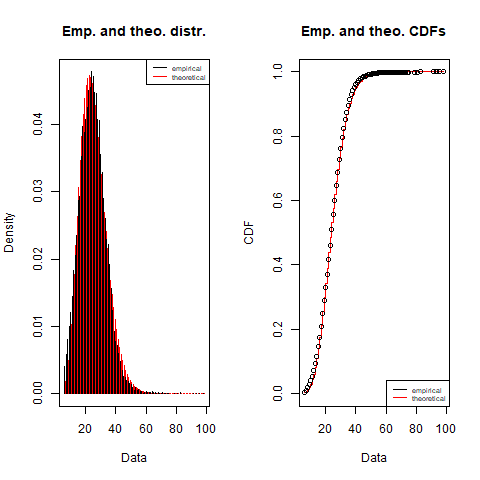

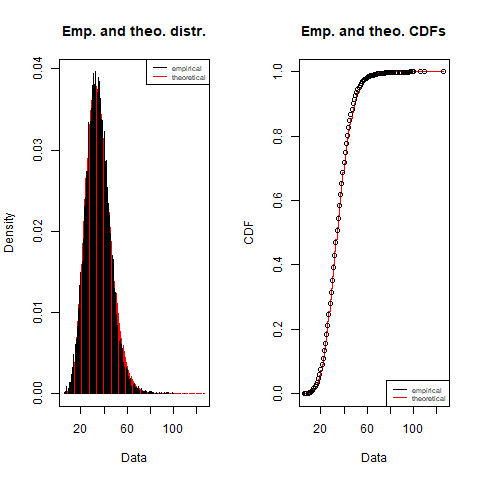


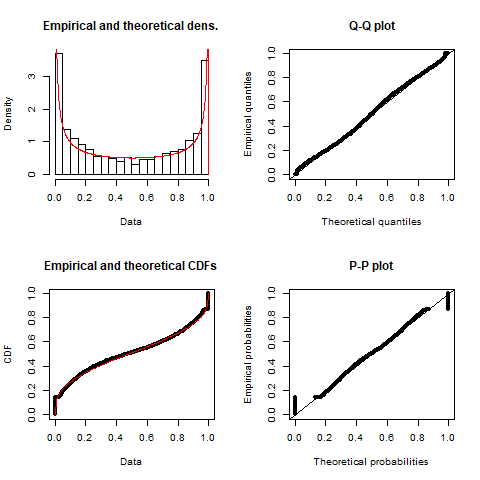

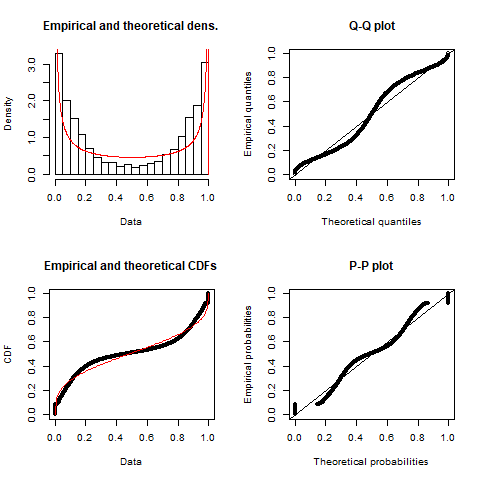


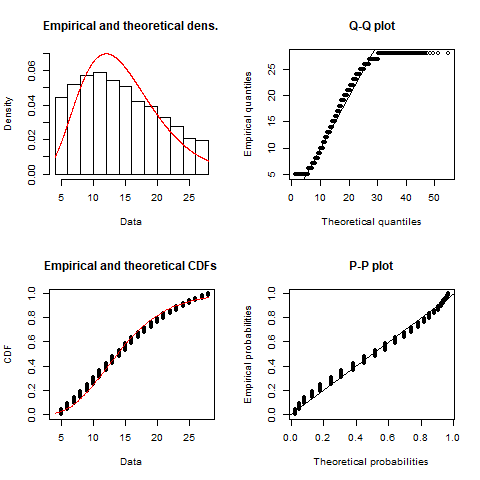

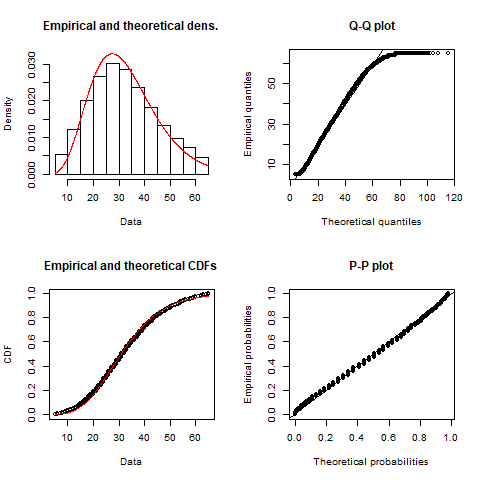


**c**


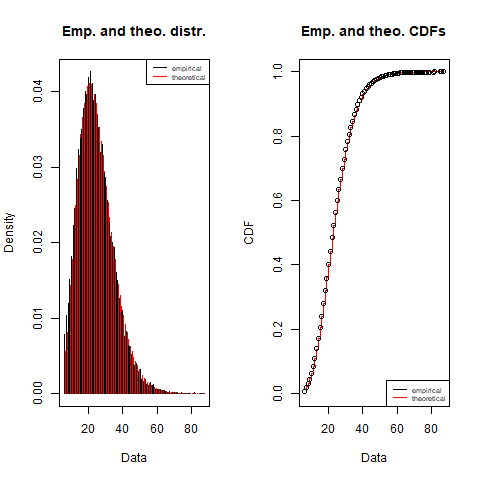

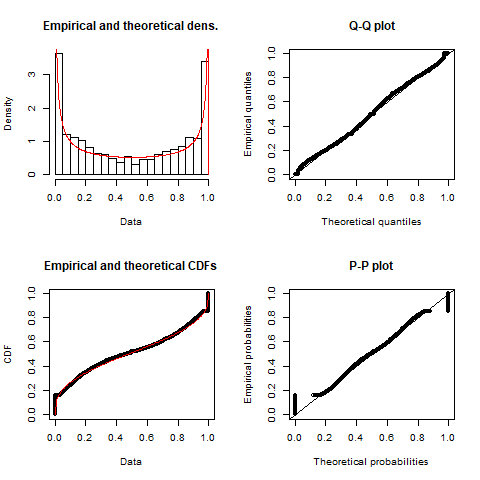

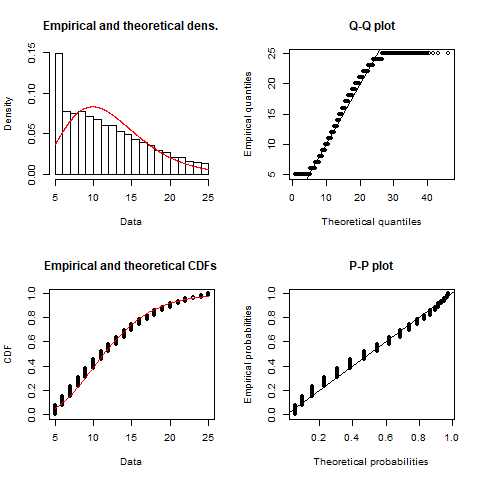

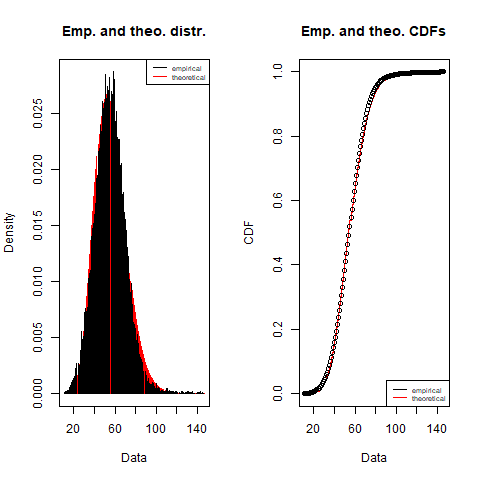


**d**


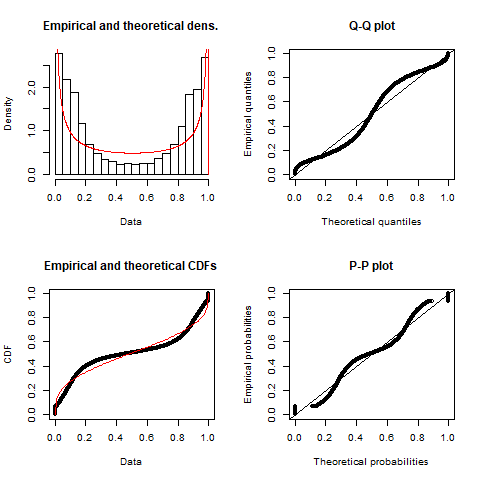

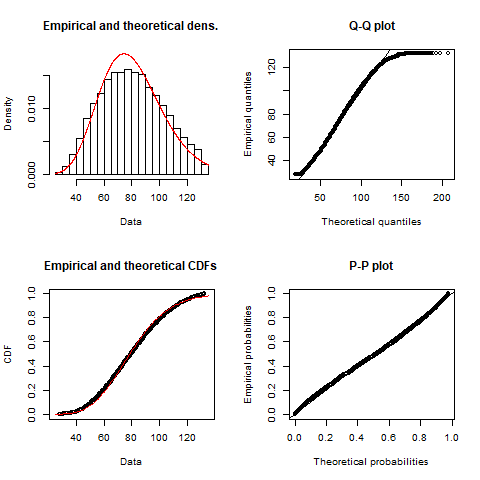


**e**

**f**


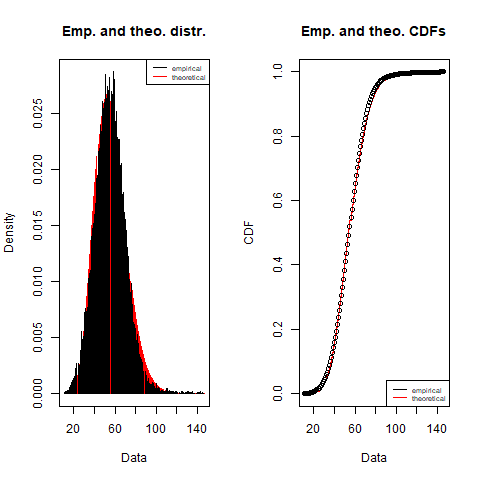

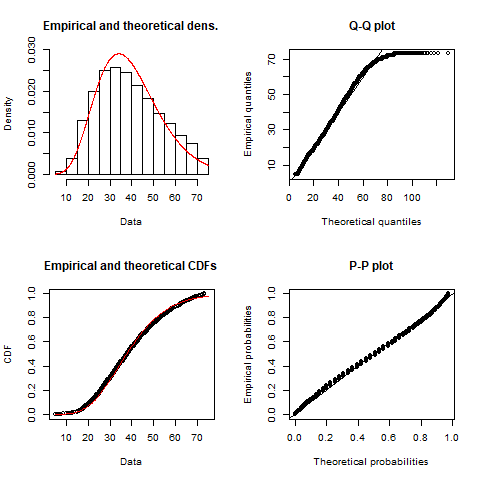

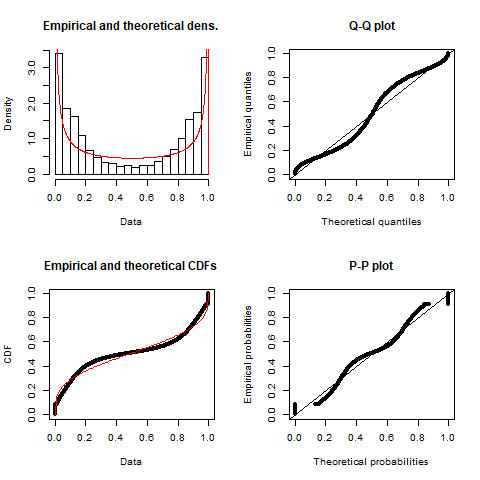

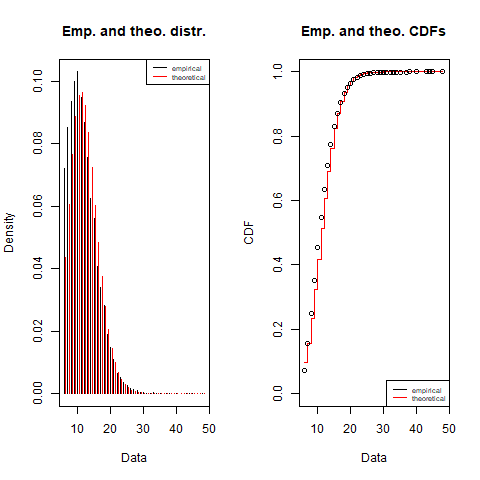

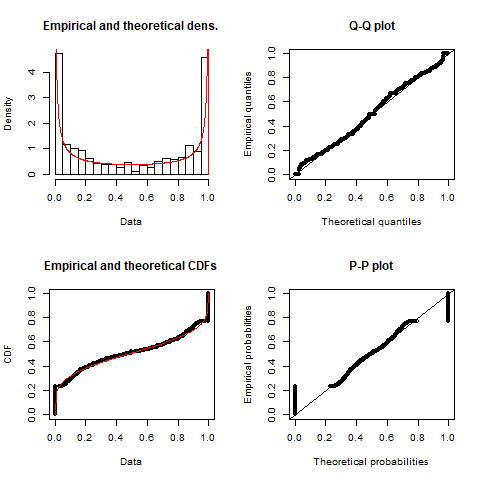

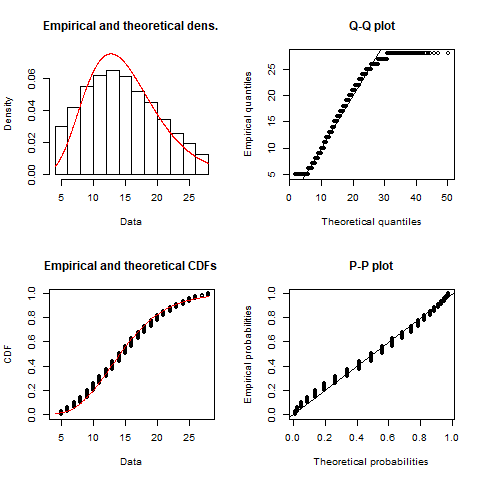


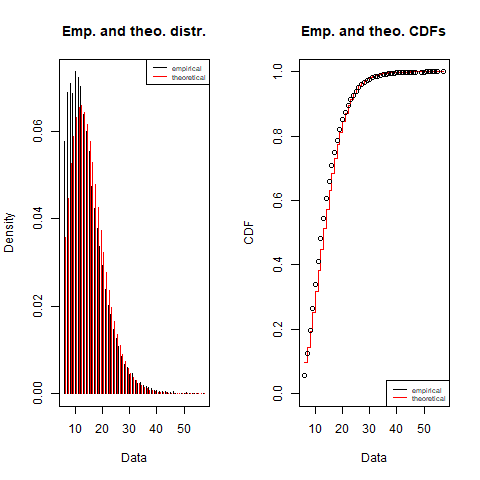


**g**


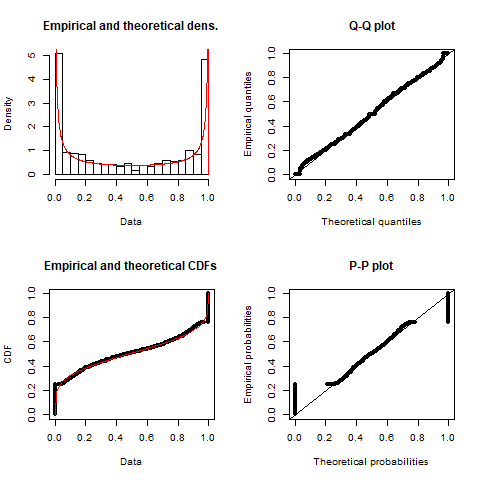


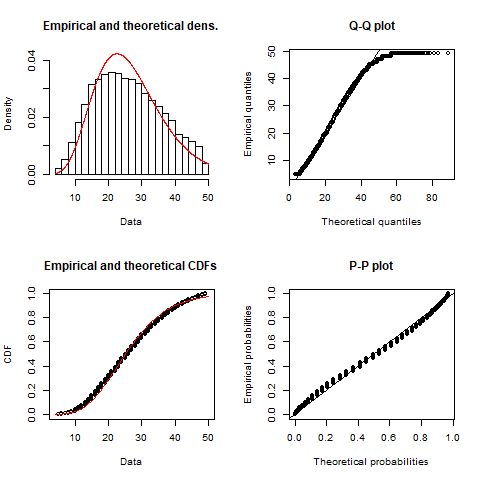


# Supplementary Figure 4 : Linear regression between negative binomial distribution parameters *µ* and *θ*.

Estimates from the fitting between a negative binomial distribution and genomic depth of coverage (see Figure S2). In blue the regression curve, in grey the 95% confidence interval.

*y* = 0.09*x* + 11.34


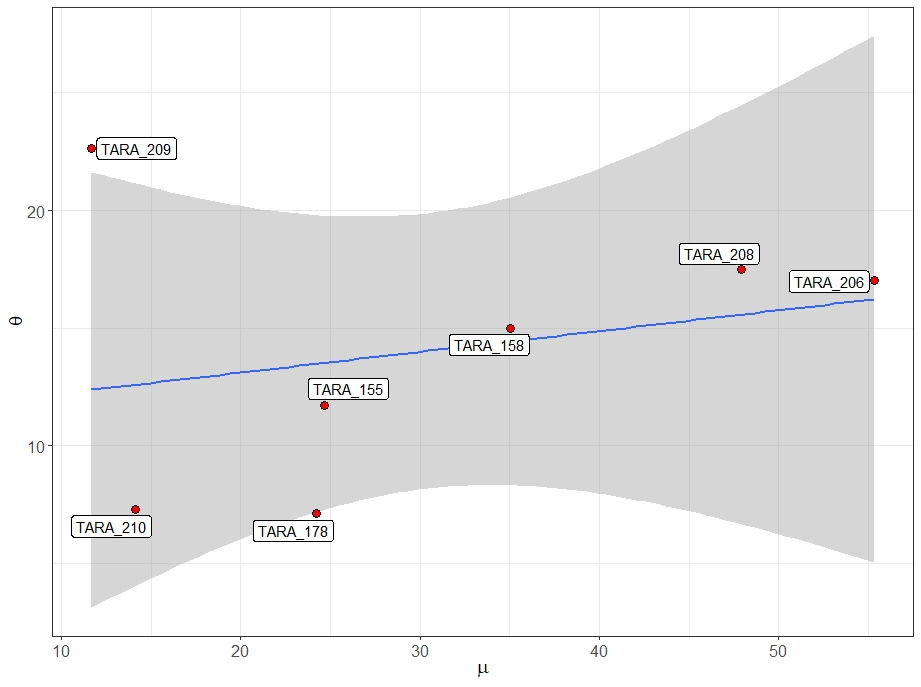


# Supplementary Figure 5 : Distributions of p-values from psADE detection performed on simulated and empirical data.

Simulations performed by generating 50,000 loci. Simulated and real p-values in blue and red respectively. The vertical dotted line represents the p-value cut-off of 0.05. Horizontal lines represent the the mean of density on p-values > 0.05 for simulated (blue) and real (red) data.


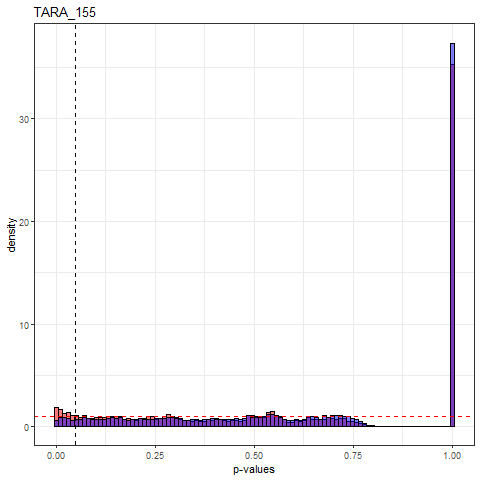

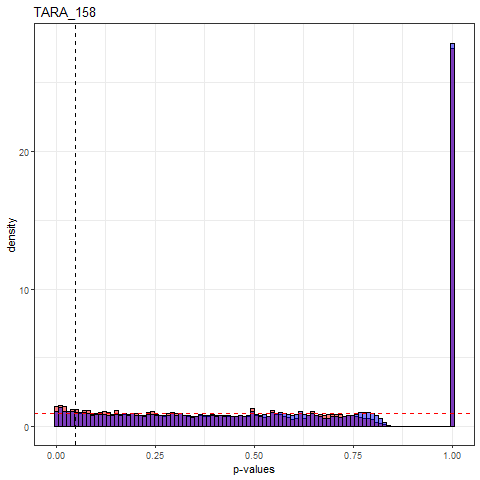


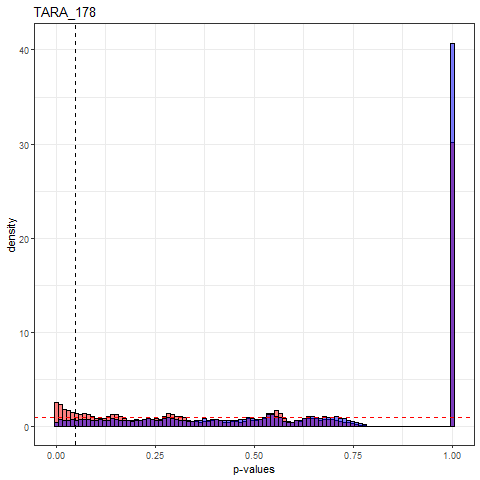

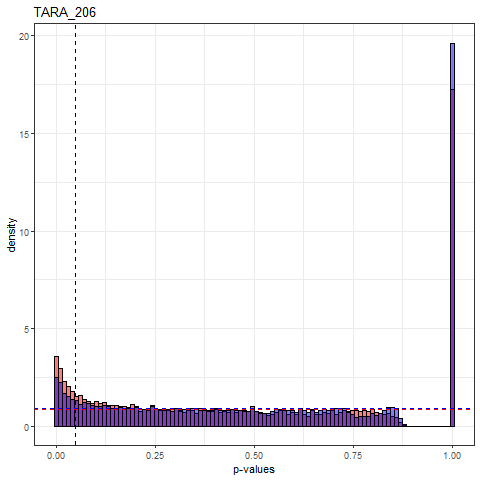


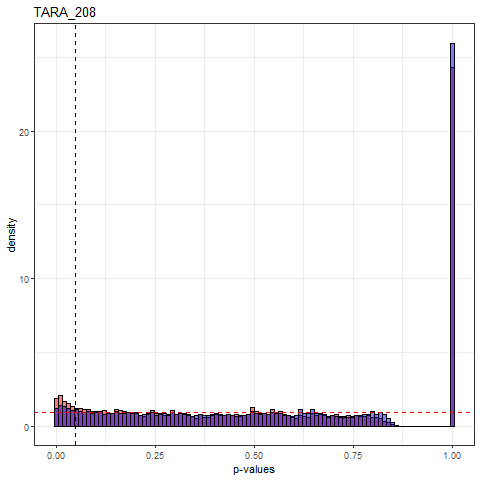

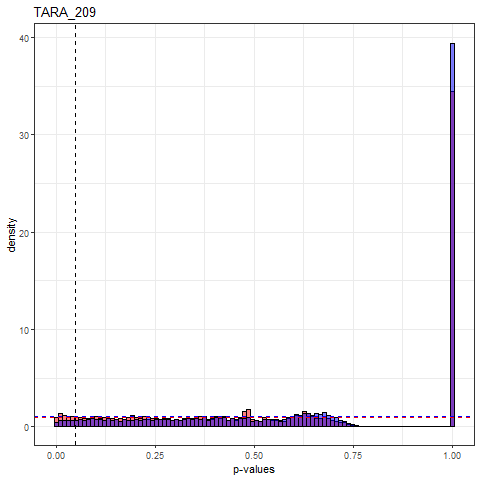


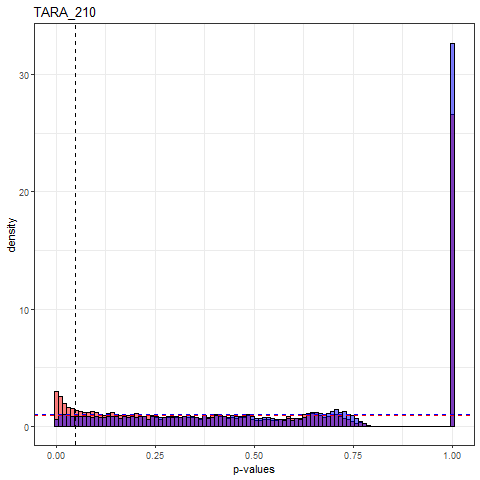


# Supplementary Figure 6 : Population genomic differentiation.

a to c, Principal Component Analysis from *pcadapt.* Each axis is a principal component with the corresponding proportion of variance explained between brackets d, Pairwise-*F*_ST_ distributions.


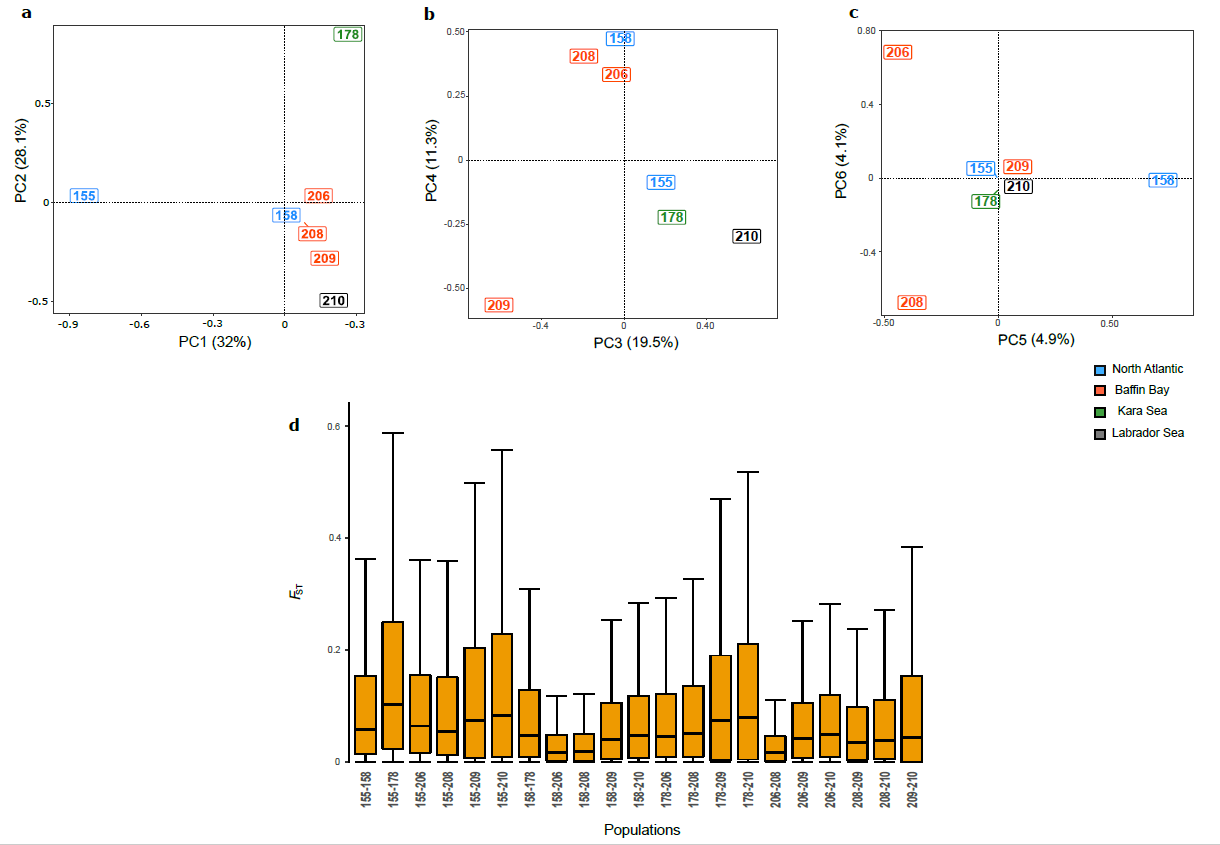


# Supplementary Figure 7 : Genomic differentiation and geographic distance.

Plot displaying Pairwise-*F*_ST_ and corresponding geographic distance. In blue, linear regression curve. In grey, the 95% confidence interval.


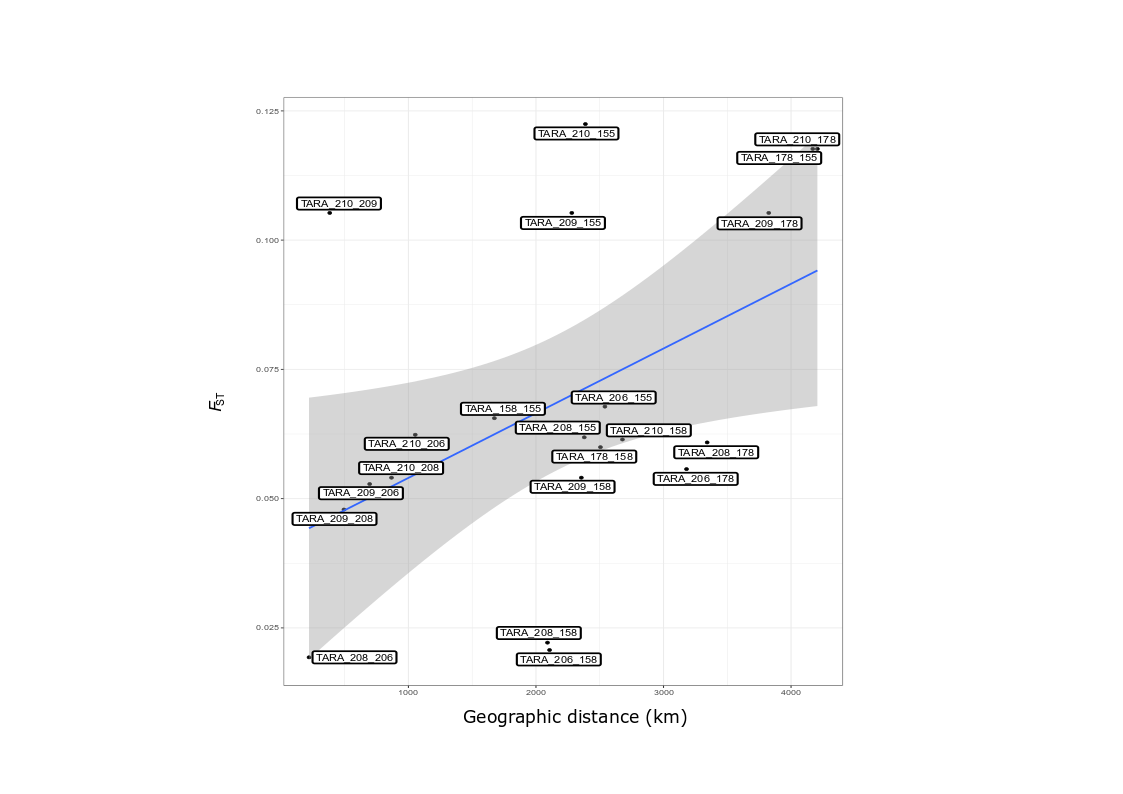


#
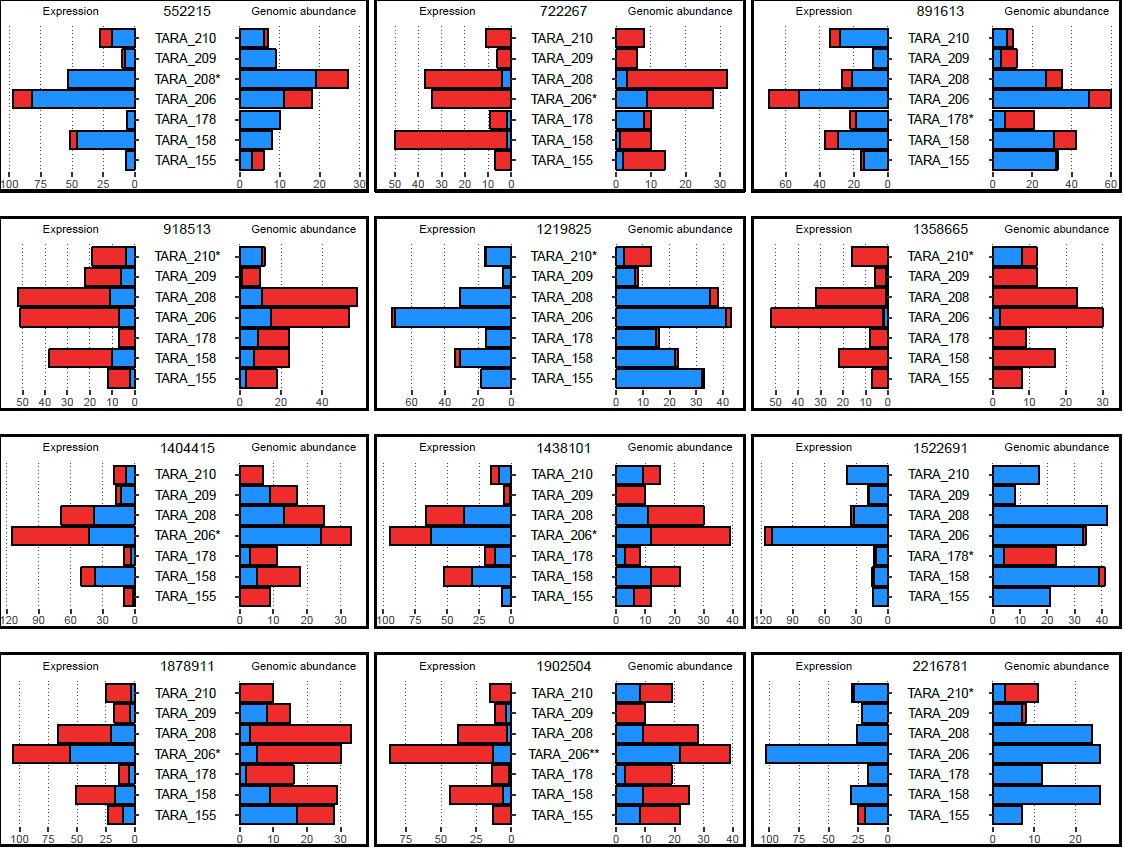
Supplementary Figure 8 : Metagenomic and metatranscriptomic profiles of candidate loci.

Each graph represents the number of read metagenomic (right) and metatranscriptomic (left) read counts in the seven population of a candidate variant under psASE and selection. Asterisks detected under psASE in the corresponding population (FTE corrected p-value < 0.05*, <0.01**, <0.001***). In blue (red), read counts of allele B (A).


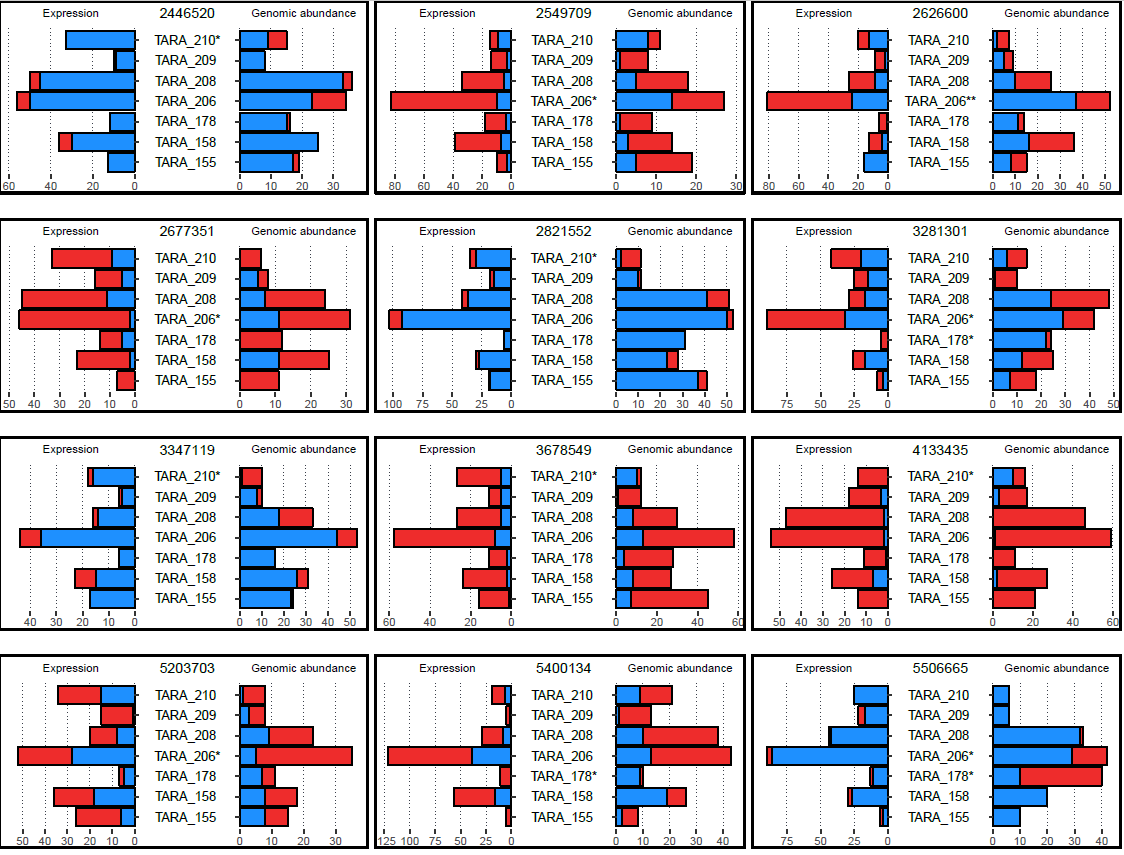

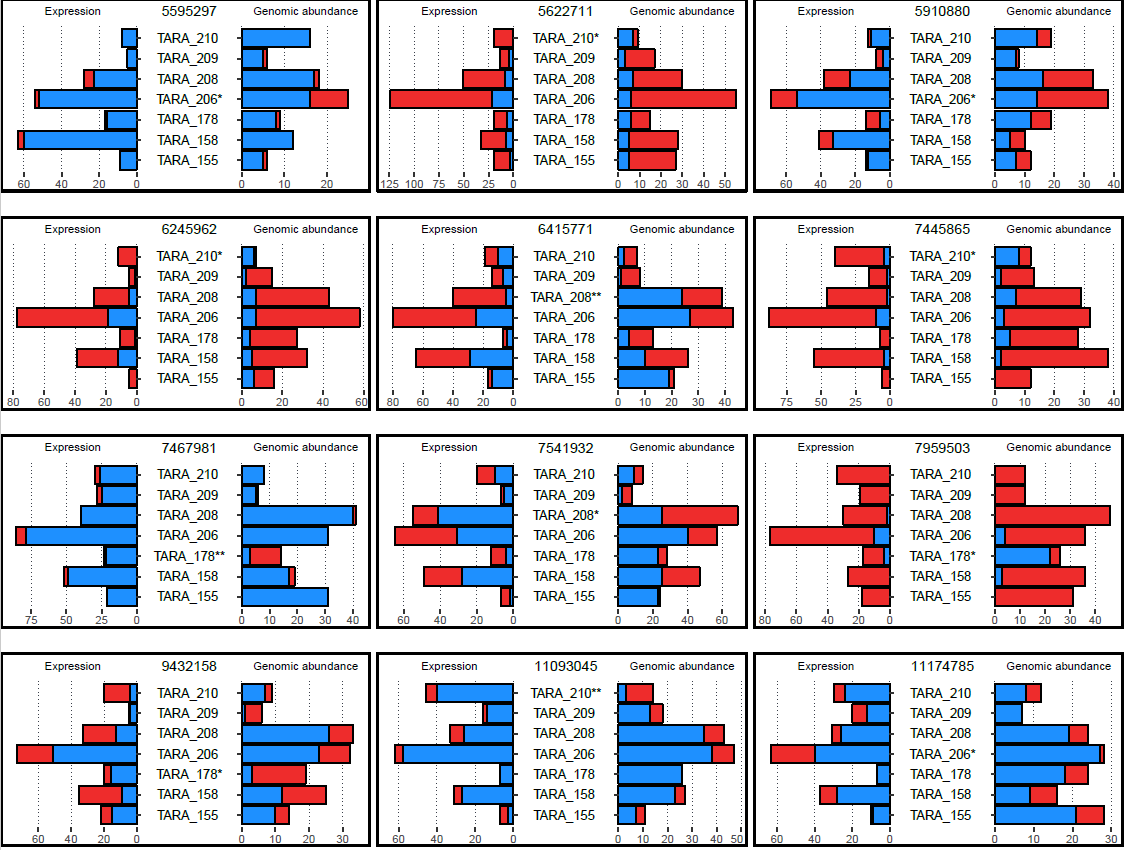


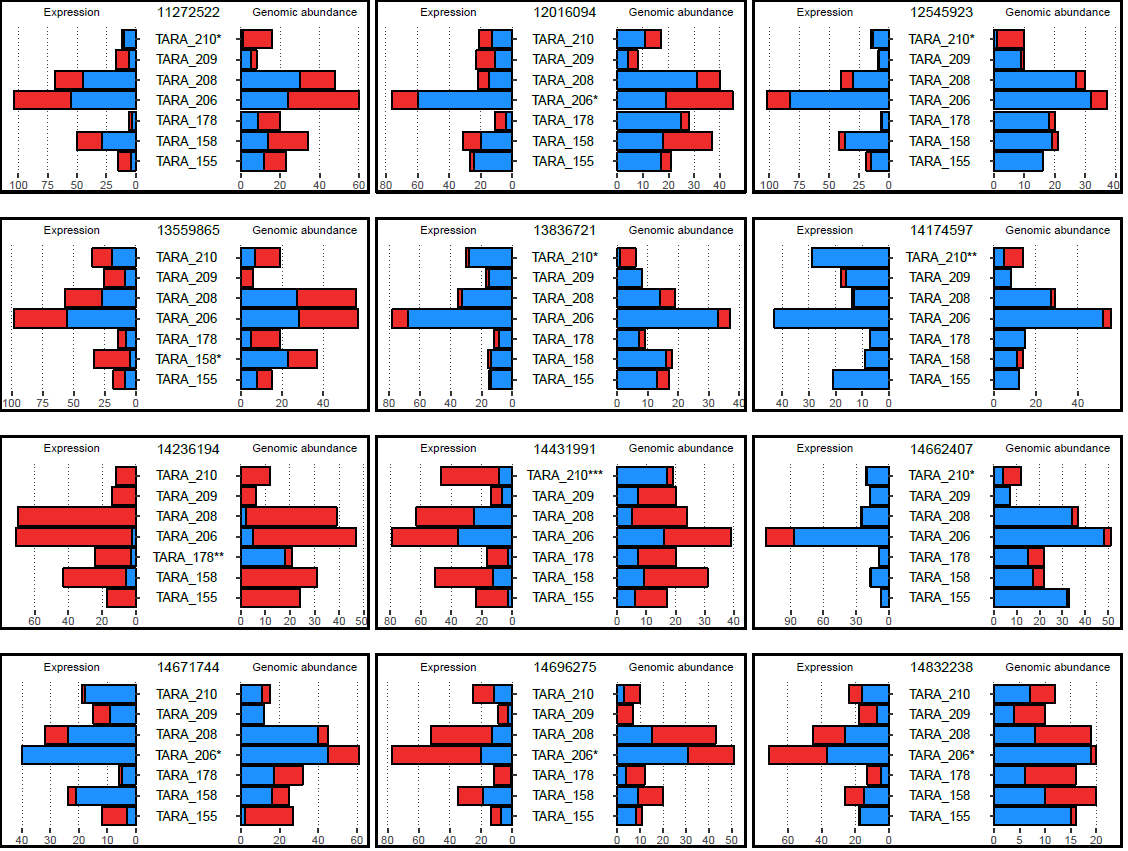

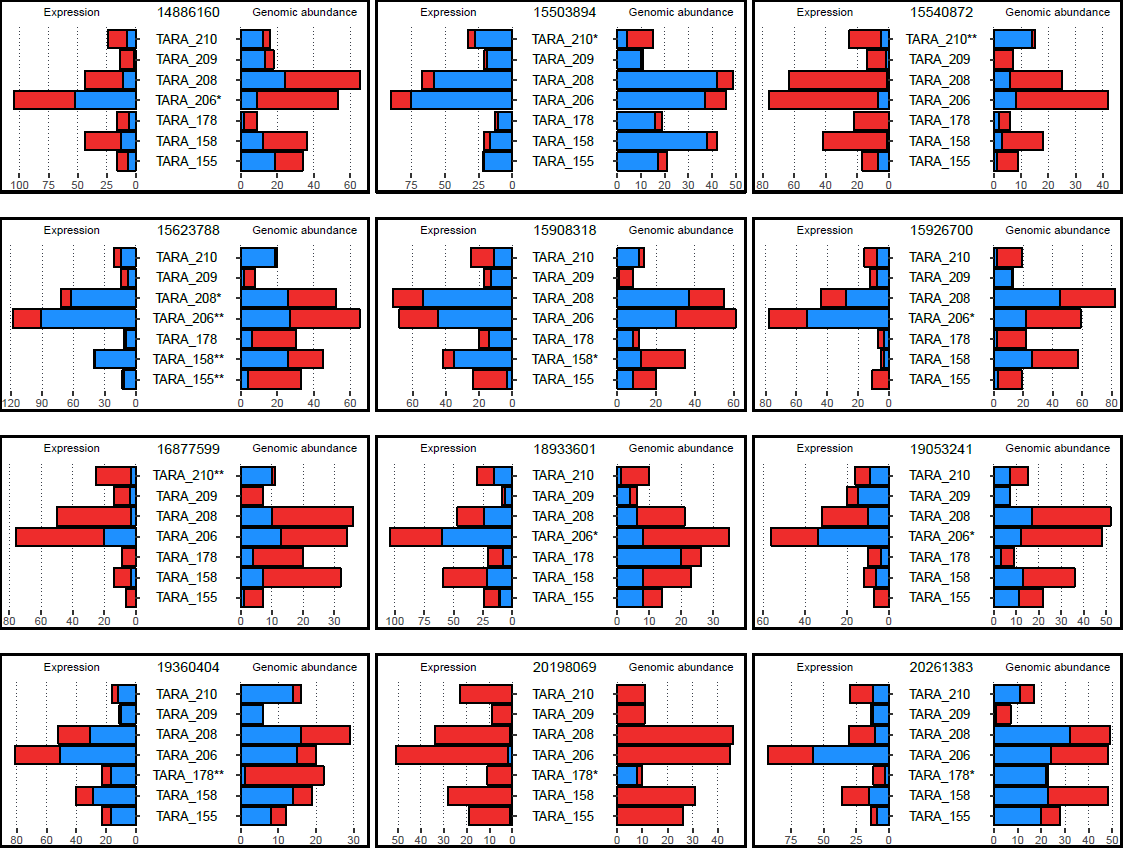


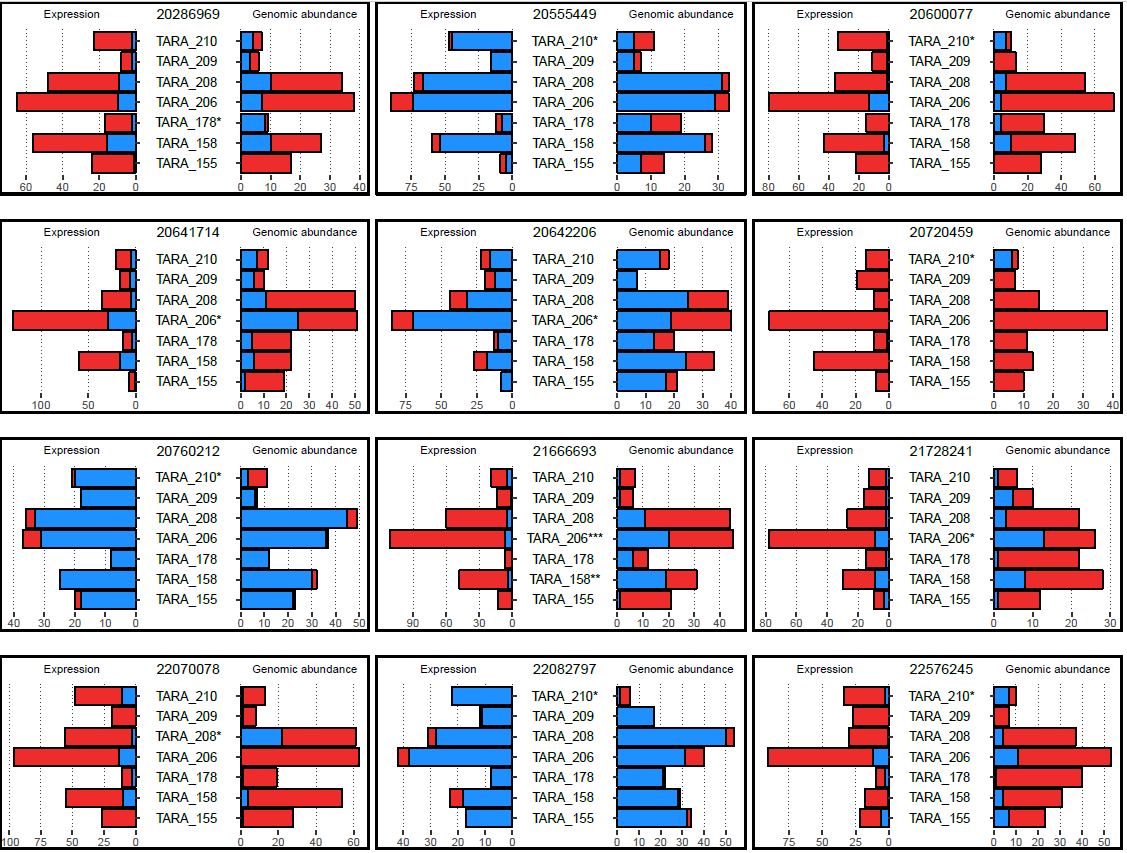

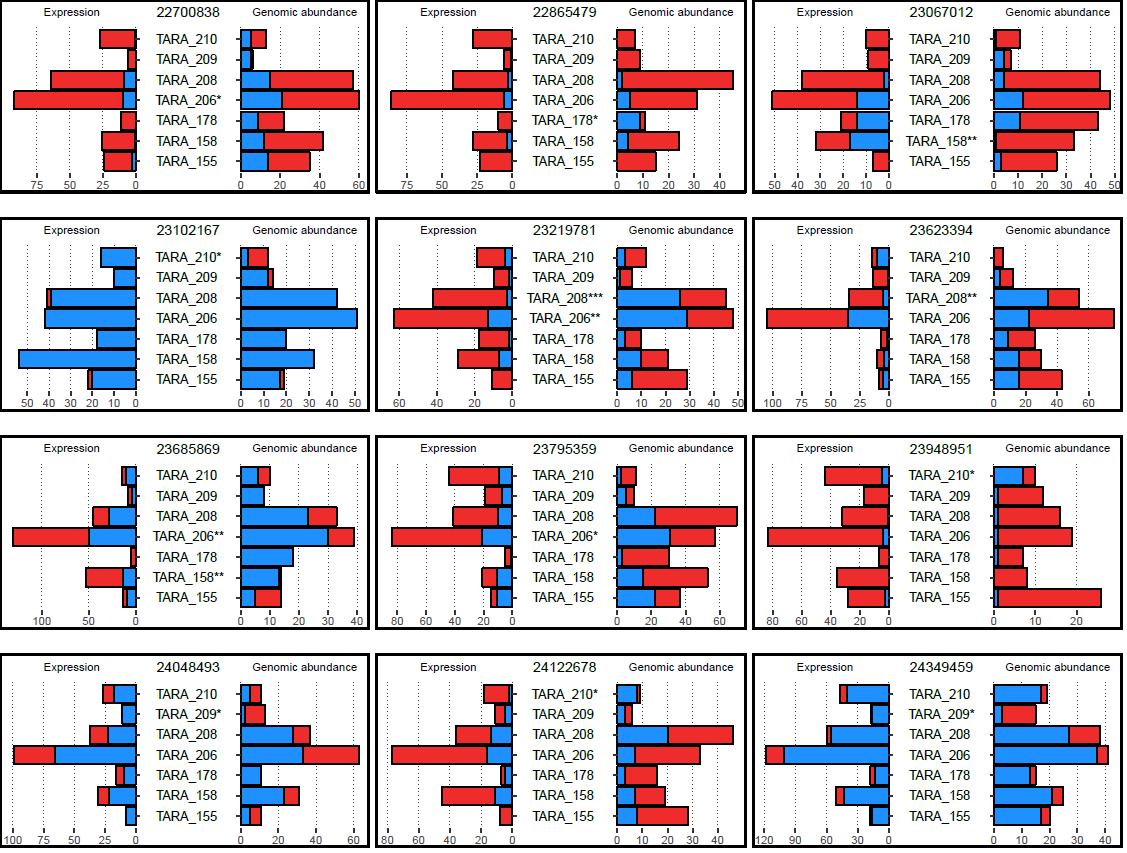


# Supplementary Figure 9 : Functional localization and effect of variants by SNPEff

Asterisks represent the significant enrichment of a category in a set of variants.


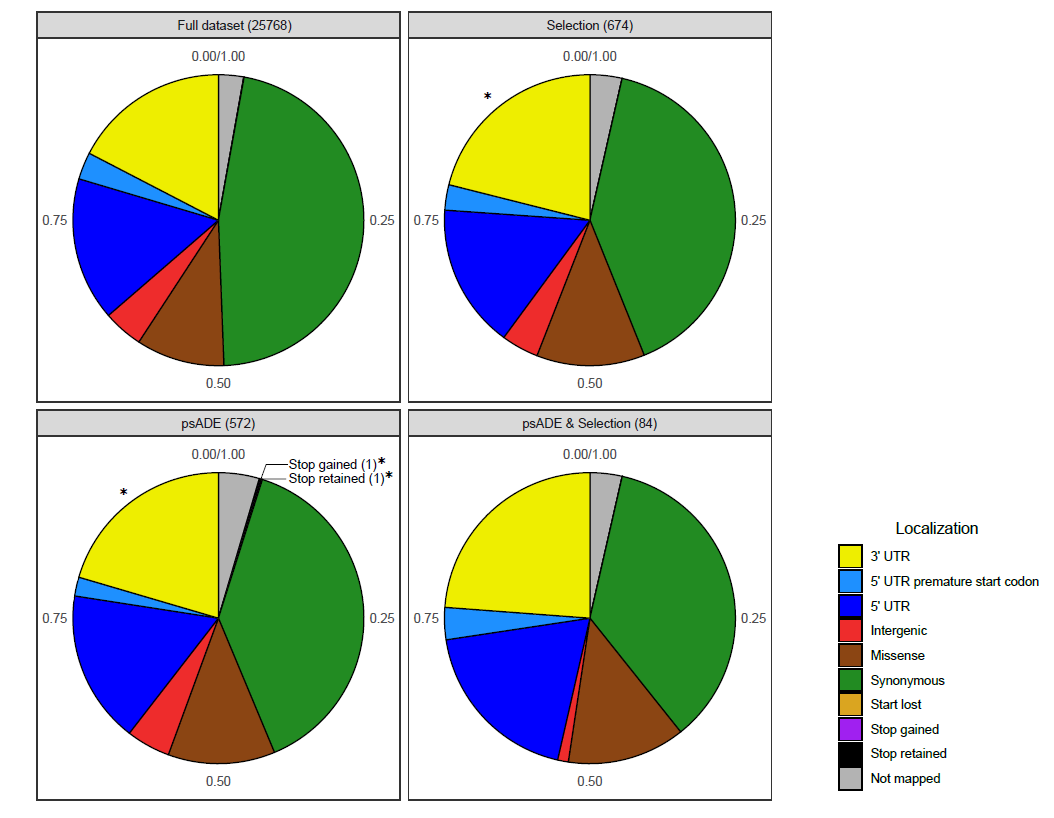


# Supplementary Figure 10 : Pfam enrichment in candidate loci

Representation of Pfam enrichment among the 84 candidate loci compared to the total set of Pfams present in the full dataset. Each dot represents a Pfam domain (x axis) and its corresponding transformed q-value. The red dots are Pfams with a significant enrichment (black dotted line, q-value <0.05), and the size corresponds to the ratio of the occurrence of a Pfam in the 84 loci on the occurrence of this Pfam in the global dataset.


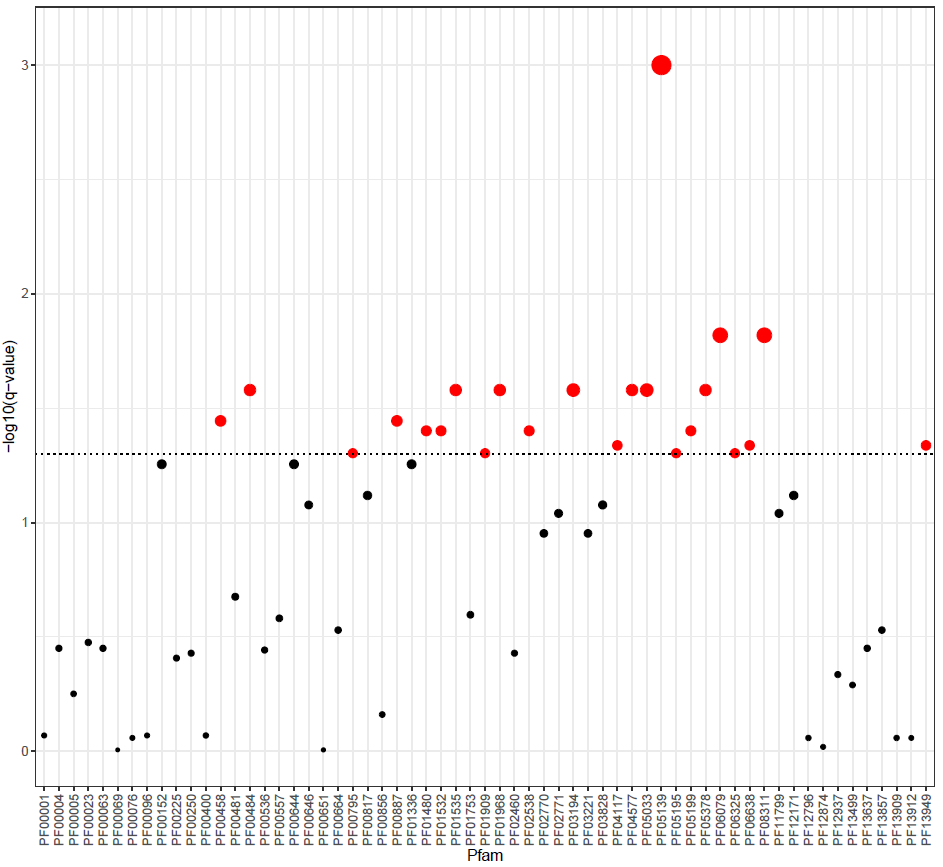


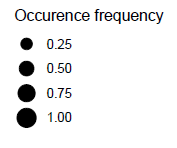


# Supplementary Table S1 : *Tara* Oceans samples and *Oithona similis* Mediterranean transcriptomes accession numbers

| **Sampling** | **Latitude** | **Longitude** | **Type** | **Description** | **Depth** | **Filter** | **Number**  **of pair of reads** | **External**  **ID** | **Submission accession** | **Study**  **accession** | **Sample**  **accession** | **Experiment**  **accession** | **Run**  **accession** |
| --- | --- | --- | --- | --- | --- | --- | --- | --- | --- | --- | --- | --- | --- |
| *Tara* Oceans | 54,5305 | -16,9377 | Metagenomics | TARA_155 | SUR | 20-180µm | 182,925,026 | SAMEA6864587 | ERA2652122 | - | ERS4592162 | ERX4145216 | ERR4181794 |
| *Tara* Oceans | 54,5305 | -16,9377 | Metatranscriptomics | TARA_155 | SUR | 20-180µm | 179,585,384 | SAMEA6864574 | ERA2652118 | - | ERS4592149 | ERX4145203 | ERR4181781 |
| *Tara* Oceans | 67,1675 | 0,2177 | Metagenomics | TARA_158 | SUR | 20-180µm | 138,512,807 | SAMEA6864586 | ERA2652122 | - | ERS4592161 | ERX4145215 | ERR4181793 |
| *Tara* Oceans | 67,1675 | 0,2177 | Metatranscriptomics | TARA_158 | SUR | 20-180µm | 168,629,428 | SAMEA6864575 | ERA2652118 | - | ERS4592150 | ERX4145204 | ERR4181782 |
| *Tara* Oceans | 77,1604 | 73,2057 | Metagenomics | TARA_178 | SUR | 20-180µm | 207,442,505 | SAMEA6864581 | ERA2652122 | - | ERS4592156 | ERX4145210 | ERR4181788 |
| *Tara* Oceans | 77,1604 | 73,2057 | Metatranscriptomics | TARA_178 | SUR | 20-180µm | 170,190,939 | SAMEA6864576 | ERA2652118 | - | ERS4592151 | ERX4145205 | ERR4181783 |
| *Tara* Oceans | 70,9618 | -53,603 | Metagenomics | TARA_206 | SUR | 20-180µm | 223,300,333 | SAMEA6864582 | ERA2652122 | - | ERS4592157 | ERX4145211 | ERR4181789 |
| *Tara* Oceans | 70,9618 | -53,603 | Metatranscriptomics | TARA_206 | SUR | 20-180µm | 189,447,541 | SAMEA6864577 | ERA2652118 | - | ERS4592152 | ERX4145206 | ERR4181784 |
| *Tara* Oceans | 69,1136 | -51,5086 | Metagenomics | TARA_208 | SUR | 20-180µm | 223,058,371 | SAMEA6864583 | ERA2652122 | - | ERS4592158 | ERX4145212 | ERR4181790 |
| *Tara* Oceans | 69,1136 | -51,5086 | Metatranscriptomics | TARA_208 | SUR | 20-180µm | 184,221,525 | SAMEA6864578 | ERA2652118 | - | ERS4592153 | ERX4145207 | ERR4181785 |
| *Tara* Oceans | 64,7127 | -53,0106 | Metagenomics | TARA_209 | SUR | 20-180µm | 181,915,803 | SAMEA6864584 | ERA2652122 | - | ERS4592159 | ERX4145213 | ERR4181791 |
| *Tara* Oceans | 64,7127 | -53,0106 | Metatranscriptomics | TARA_209 | SUR | 20-180µm | 178,106,374 | SAMEA6864579 | ERA2652118 | - | ERS4592154 | ERX4145208 | ERR4181786 |
| *Tara* Oceans | 61,5442 | -55,9865 | Metagenomics | TARA_210 | SUR | 20-180µm | 182,303,005 | SAMEA6864585 | ERA2652122 | - | ERS4592160 | ERX4145214 | ERR4181792 |
| *Tara* Oceans | 61,5442 | -55,9865 | Metatranscriptomics | TARA_210 | SUR | 20-180µm | 179,419,896 | SAMEA6864580 | ERA2652118 | - | ERS4592155 | ERX4145209 | ERR4181787 |
| Toulon | 43,1001 | 5,9482 | Transcriptome | Copepodite 1 | 0-10m | 90-200µm | 17,823,526 | SAMEA5540033 | ERA1801976 | ERP114539 | ERS3342094 | ERX3282627 | ERR3255855 |
| Toulon | 43,1001 | 5,9482 | Transcriptome | Copepodite 2 | 0-10m | 90-200µm | 19,237,768 | SAMEA5540033 | ERA1801976 | ERP114539 | ERS3342094 | ERX3282628 | ERR3255856 |
| Toulon | 43,1001 | 5,9482 | Transcriptome | Copepodite 3 | 0-10m | 90-200µm | 18,292,442 | SAMEA5540033 | ERA1801976 | ERP114539 | ERS3342094 | ERX3282629 | ERR3255857 |
| Toulon | 43,1001 | 5,9482 | Transcriptome | Copepodite 4 | 0-10m | 90-200µm | 13,933,841 | SAMEA5540033 | ERA1801976 | ERP114539 | ERS3342094 | ERX3282630 | ERR3255858 |
| Toulon | 43,1001 | 5,9482 | Transcriptome | Male 1 | 0-10m | 90-200µm | 18,052,409 | SAMEA5540033 | ERA1801976 | ERP114539 | ERS3342094 | ERX3282631 | ERR3255859 |
| Toulon | 43,1001 | 5,9482 | Transcriptome | Male 2 | 0-10m | 90-200µm | 21,048,656 | SAMEA5540033 | ERA1801976 | ERP114539 | ERS3342094 | ERX3282632 | ERR3255860 |
| Toulon | 43,1001 | 5,9482 | Transcriptome | Male 3 | 0-10m | 90-200µm | 19,129,821 | SAMEA5540033 | ERA1801976 | ERP114539 | ERS3342094 | ERX3282633 | ERR3255861 |
| Toulon | 43,1001 | 5,9482 | Transcriptome | Male 4 | 0-10m | 90-200µm | 17,624,872 | SAMEA5540033 | ERA1801976 | ERP114539 | ERS3342094 | ERX3282634 | ERR3255862 |

# Supplementary Table S2 : *Oithona similis* Mediterranean transcriptomes summary.

|  | **Copepodite** | | | |  | **Male** | | | |
| --- | --- | --- | --- | --- | --- | --- | --- | --- | --- |
|  | **1** | **2** | **3** | **4** |  | **1** | **2** | **3** | **4** |
| **Number of pairs of reads** | 18,052,409 | 21,048,656 | 19,129,821 | 17,624,872 |  | 17,823,526 | 19,237,768 | 18,292,442 | 13,933,841 |
| **Number of transcripts**  ***(Trinity)*** | 81,802 | 82,811 | 131,747 | 83,244 |  | 117,951 | 79,938 | 115,170 | 79,038 |
| **Number of predicted ORFs *(Transdecoder)*** | 20,389 | 20,963 | 42,404 | 20,746 |  | 38,580 | 19,488 | 34,179 | 20,482 |
| **N50 (L50)** | 1,452 (4449) | 1,389 (4,849) | 1,056 (9,075) | 1,521 (4,325) |  | 882 (9,721) | 1,314 (4,267) | 942 (8,325) | 1,212 (4,842) |
| **longest length** | 10,581 | 13,698 | 14,136 | 10,245 |  | 19,128 | 10,116 | 9000 | 10,110 |
| **average length** | 1,015 | 997 | 795 | 1,038 |  | 726 | 935 | 753 | 901 |

# Supplementary Table S3 : Distribution parameters of genomic depth of coverage, variant frequency and expression level.


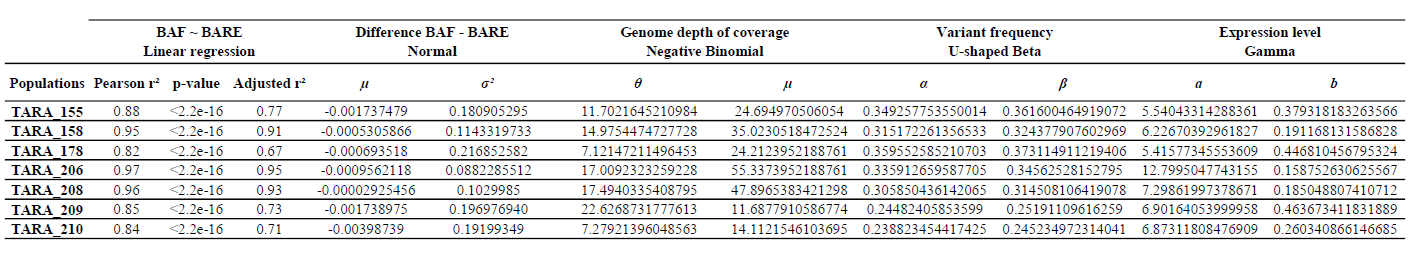


# Supplementary Table S4 : Estimation of false-positives in psADE detection.

|  | **Simulated Data** | | |  | **Real Data** | | |  |
| --- | --- | --- | --- | --- | --- | --- | --- | --- |
| **Populations** | **Number of**  **tested variants** | **Number of**  **psADE** | **Proportion** |  | **Number of**  **tested variants** | **Number of**  **psADE** | **Proportion** | **True-positive**  **proportion** |
| **TARA_155** | 31,749 | 0 | 0 |  | 18,812 | 36 | 0.00191 | 1 |
| **TARA_158** | 32,252 | 1 | 0.00003 |  | 21,476 | 53 | 0.00247 | 0.98 |
| **TARA_178** | 31,887 | 0 | 0 |  | 18,145 | 86 | 0.00474 | 1 |
| **TARA_206** | 35,565 | 138 | 0.00388 |  | 22,578 | 296 | 0.01311 | 0.7 |
| **TARA_208** | 33,414 | 4 | 0.00120 |  | 21,469 | 70 | 0.00326 | 0.96 |
| **TARA_209** | 20,994 | 0 | 0 |  | 13,956 | 9 | 0.00064 | 1 |
| **TARA_210** | 21,737 | 0 | 0 |  | 13,454 | 111 | 0.00825 | 1 |
